# Stochastic Dynamics of Interacting Haematopoietic Stem Cell Niche Lineages

**DOI:** 10.1371/journal.pcbi.1003794

**Published:** 2014-09-04

**Authors:** Tamás Székely, Kevin Burrage, Marc Mangel, Michael B. Bonsall

**Affiliations:** 1 Department of Computer Science, University of Oxford, Oxford, United Kingdom; 2 Department of Mathematics, Queensland University of Technology, Brisbane, Queensland, Australia; 3 Department of Applied Mathematics and Statistics, University of California Santa Cruz, Santa Cruz, California, United States of America; 4 Department of Biology, University of Bergen, Bergen, Norway; 5 Mathematical Ecology Research Group, Department of Zoology, University of Oxford, Oxford, United Kingdom; ATP-Group, Portugal

## Abstract

Since we still know very little about stem cells in their natural environment, it is useful to explore their dynamics through modelling and simulation, as well as experimentally. Most models of stem cell systems are based on deterministic differential equations that ignore the natural heterogeneity of stem cell populations. This is not appropriate at the level of individual cells and niches, when randomness is more likely to affect dynamics. In this paper, we introduce a fast stochastic method for simulating a metapopulation of stem cell niche lineages, that is, many sub-populations that together form a heterogeneous metapopulation, over time. By selecting the common limiting timestep, our method ensures that the entire metapopulation is simulated synchronously. This is important, as it allows us to introduce interactions between separate niche lineages, which would otherwise be impossible. We expand our method to enable the coupling of many lineages into niche groups, where differentiated cells are pooled within each niche group. Using this method, we explore the dynamics of the haematopoietic system from a demand control system perspective. We find that coupling together niche lineages allows the organism to regulate blood cell numbers as closely as possible to the homeostatic optimum. Furthermore, coupled lineages respond better than uncoupled ones to random perturbations, here the loss of some myeloid cells. This could imply that it is advantageous for an organism to connect together its niche lineages into groups. Our results suggest that a potential fruitful empirical direction will be to understand how stem cell descendants communicate with the niche and how cancer may arise as a result of a failure of such communication.

## Author Summary

Stem cells portend great potential for advances in medicine. However, these advances require detailed understanding of the dynamics of stem cells. *In vitro* studies are now routine and challenge our preconceptions about stem cell biology, but the dynamics of stem cells *in vivo* remain poorly understood. Thus, there is a real need for novel computational frameworks for general understanding and predictions about experiments on stem cells in their native environments. By implementing a stochastic model of stem cell dynamics, generically based on the bone marrow system, in a novel, fast and computationally efficient way, we show how different couplings of stem cell niche lineages lead to different predictions about homeostatic control. Understanding the demand control of stem cell systems is essential to both predicting *in vivo* stem cell dynamics and also how its breakdown may lead to the development of cancers of the blood system.

## Introduction

Stem cells offer exciting potential for regenerative therapy, with ultimate possibilities being the ability to regenerate limbs and heal genetic diseases [1], [2]. Although studies have begun to address these issues, much work remains to be done [3], [4]. Indeed, much of our knowledge of stem cells is derived from *in vitro* experiments, where the stem cells have been relocated from their native environment. For instance, in haematopoietic (blood-producing) stem cell experiments the stem cells are often isolated from a donor, expanded *in vitro*, and transplanted into a lethally irradiated host, with the question of interest being how the stem cells respond to this new environment (e.g., [5]). However, it is difficult to draw conclusions about the role and behaviour of stem cells *in vivo*, when experimentally we must investigate them in foreign environments [6], [7]. Thus, theoretical models of stem cell systems are valuable tools, allowing us to think about stem cells in their native environments when this cannot yet be done experimentally.

*In vivo*, stem cells are generally found in special microenvironments, or niches, which are defined by a complex set of biochemical and physical conditions that feed back on each other [2], [8]. Niches play a critical role in the function and behaviour of stem cells [2], [9]. For instance, experimentally changing certain niche attributes affects the dynamics of the stem cells inside them [10]. In addition, stem cells are often not single entities that exist independently of each other, but instead form an interacting population that includes stem cells and their more differentiated products, both within and outside the niche [11], [12]. Moreover, even separate niches can affect each other, for instance through the effects of their daughter cells or migration (e.g., [13]).

We focus on modelling the haematopoietic stem cell (HSC) system, for two reasons. Firstly, it is probably the most well-characterised stem cell system; secondly, it is representative of stem cell systems in general, incorporating their essential properties such as self-renewal, differentiation, multiple lineage choices and feedbacks to regulate cell populations [9], [14]. This allows us to start thinking about heterogeneity and the introduction of population interactions in a comparatively simple setting [15]. It seems that there are a minimum of two distinct niche types in bone marrow, although their relationship to each other is not fully clear, nor has their connection to the different primitive cell types been unambiguously elucidated [16]–[20]. Spatially, the HSCs themselves are spread throughout the bone marrow (as well as certain other organs, such as the liver and spleen), each in its own individual ‘facultative niche’ [17], [21]–[24]. To be precise in our definition, henceforth we refer only to these facultative niches as ‘niches’. Bone marrow thus contains an entire population of niches, with each niche containing small numbers of HSCs, and these HSCs can differentiate into blood cells, which eventually join the bloodstream.

The HSC system operates by demand control [25]: there is a target level of differentiated blood cells, the homeostatic level, which is set by natural selection [15], [26], [27], and which the organism attains by differentiation of the HSCs and blood progenitor cells into appropriate differentiated blood cell types [27], [28]. This seems to be achieved by feedback from the differentiated progeny of the HSCs in the bloodstream [28]–[30]. In addition, there is also feedback from differentiated progeny that have not entered the bloodstream, but remain localised to the niche [12]. The HSC system must respond rapidly to perturbations such as wounding or infection, and even under normal conditions the blood cell turnover of an average human being is around one trillion cells per day [31]. Such enormous numbers mean that it is important to have a robust feedback mechanism for proper functioning of the system.

The complex nature of the HSC system, with different blood cell types and feedbacks, as well as many spatially separate niches, means that it is difficult to model. In general, current models of stem cell dynamics involve either only one focal stem cell, or a homogeneous population of each cell type, and are modelled using ordinary differential equations (ODEs) [15]. Although such models can give useful results, it is important to include heterogeneity in the picture [32]. For example, there is considerable heterogeneity between individual stem cell clones [33], [34]; this heterogeneity is also present *within* clonal cell lines [35], [36], and was even observed many years ago by Till et al. [5], as well as by Suda et al. [37]. However, in the intervening decades the deterministic view of stem cell differentiation has taken hold with great success and has led towards understanding the feedback between differentiated and primitive cells [28], [38]. More recently there has been a shift in emphasis, with stochastic models being used to examine the dynamics and the evolution of mutations in a stem cell population [39], phenotypic equilibrium in a cancer cell population [40], and the effects of different control mechanisms on stem cell populations [41], [42].

Two of us have already proposed a population biology framework for stem cell dynamics, with the theme “stem cell biology is population biology” [15], [27]. We used an ODE model of one niche lineage to show how evolution affects the decision of whether to differentiate into myeloid or lymphoid cells. In this paper, we expand on this framework by considering the stochastic dynamics of a heterogeneous metapopulation of niche lineages, comprised of stem, progenitor and differentiated blood cells. For simplicity, we restrict our study to intrinsic heterogeneity only (that is heterogeneity arising in a clonal cell population in an identical environment). We take into account the further consideration that while the niches (containing the primitive cells) may be distinct, the blood cells are mixed in the bloodstream, and the niche lineages could be controlled by feedback from the entire bloodstream rather than just their own, possibly localised, descendants. Thus we *couple together* separate niche lineages, allowing them to interact with each other through their differentiated progeny. Our main aims in this paper are to 1) establish the stochastic framework, 2) investigate the dynamics of the stochastic system, 3) explore how coupling niche lineages together into niche groups affects the system dynamics, and 4) whether it has any effect on the response of the entire system to a perturbation.

We first develop the stochastic modelling framework. Since stochastic simulations can be slow, we introduce a fast, approximate method for simulating an entire metapopulation of HSC niche lineages. We then describe how to take into account the interactions (feedbacks) from the differentiated blood cells on to the primitive cells in the niche (stem and progenitor cells) in our simulations. We simulate a metapopulation of lineages through time, which first settles to homeostasis and is then perturbed by reducing blood cell numbers. After the perturbation, there is a peak in blood cell numbers as the stem and progenitor cells replenish them. We investigate the effects of coupling niche lineages together: that is, what happens when the feedbacks are averaged across many niche lineages (the number of niches averaged over is called the ‘niche group size’). We find that 1) coupling niche lineages shifts the mean cell populations at steady state, and changes the shape of the cells’ distributions; 2) as more lineages are coupled together, the total blood cells in each coupled niche group approach the target steady state of the system; 3) different perturbation types elicit a different response from the system, and when blood cells are perturbed randomly, niche lineages coupled into larger groups respond better than smaller groups and uncoupled lineages. Taken together, these results imply that for the organism, connecting the individual niche lineages into larger niche groups is advantageous, both for optimal regulation of the overall system and for responding to random perturbations.

## Methods

### HSC Model

**Figure 1:**
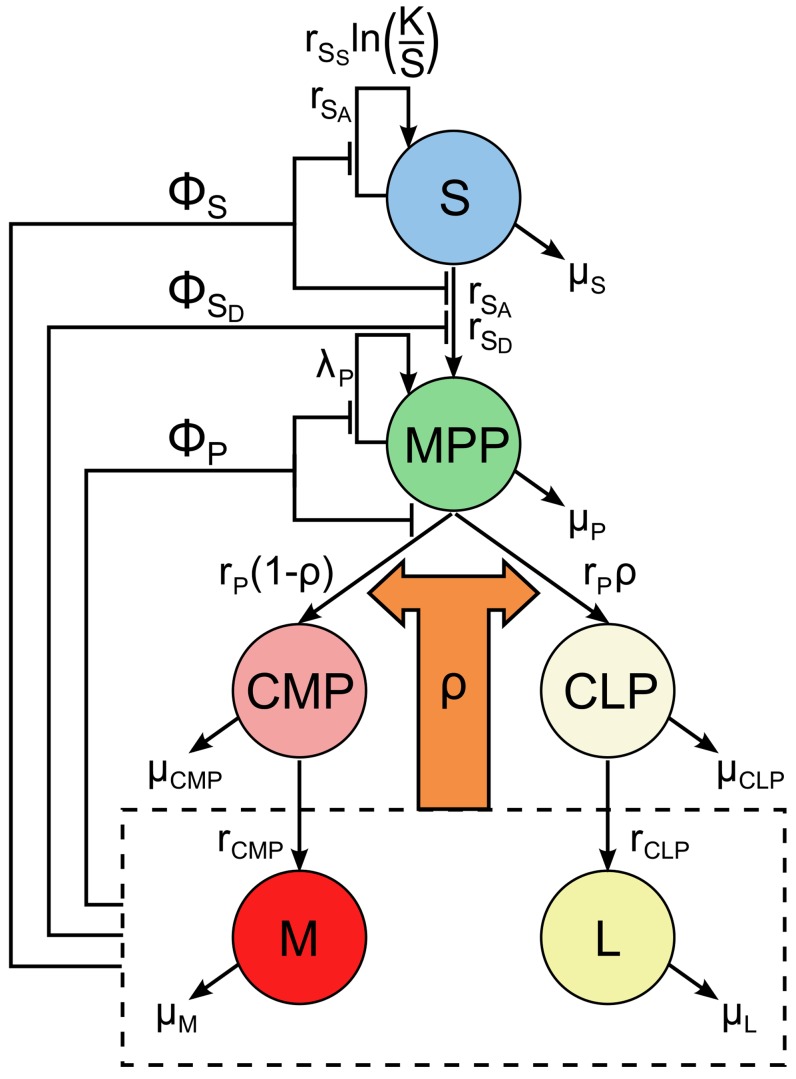
One niche lineage of the stochastic system, with all state transitions and feedbacks shown. Functions 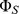, 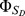 and 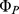 are feedbacks on to the activity of 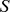, differentiation rate of 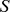 and activity of 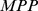, respectively, and 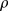 is the so-called MPCR, which determines the probability of an 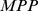 transitioning to either the lymphoid or myeloid lineages, and is defined in Eq. (2).

We begin with the model of the HSC system as developed by Mangel and Bonsall [27], which characterises the stem cell niche and its products as a control system driven ultimately by demand from the organism (Fig. 1). The system consists of a HSC niche, containing stem and progenitor cells, and its fully differentiated progeny cells in the bloodstream. The demand from the organism occurs via changes in the levels of differentiated blood cells, which feed back this demand to the primitive (stem and progenitor) cells.

Specifically, the model is comprised of the populations of stem cells (*S*), multipotent progenitor cells (*MPP*), common lymphoid and common myeloid progenitor cells (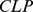 and 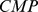, respectively) and their fully differentiated products, lymphoid and myeloid blood cells (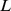 and 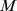, respectively). Although there are many differentiated blood cell types (see, for example, [14]), here we classify them as myeloid and lymphoid types for the sake of simplicity. Thus our model has six state variables, to correspond to the population of each cell type, with certain transitions allowed between the states: 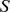 self-renewal via either symmetric or asymmetric division; 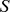 (symmetric) differentiation; 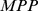 multiplication or differentiation into 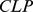 or 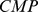, i.e. either the lymphoid or myeloid route, with relative probabilities 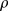 and 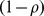, respectively (see below); 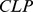 and 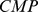 differentiation into 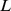 or 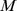, respectively; in addition, all cell types can die. In [27], these transitions are written down as a set of ODEs (also given in Supporting Text S1, Section 1), which give the rate of change of each state in time as a function of the current state. Here, we use the stochastic version of this model, given by formulae for each transition between the states, which occur probabilistically (Table 1).

**Table 1:** Transitions in the stochastic model.

#	Transition	Transition propensity	Process
1	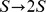	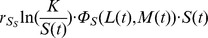	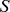 symmetric division (self-renewal)
2	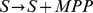	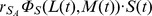	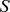 asymmetric division (self-renewal)
3	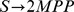	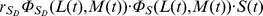	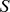 symmetric differentiation
4	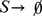	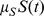	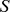 death
5	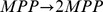	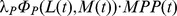	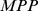 renewal
6	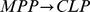	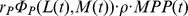	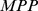 differentiation to 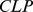
7	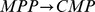	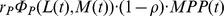	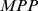 differentiation to 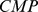
8	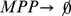	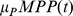	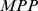 death
9	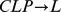	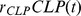	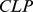 differentiation
10	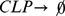	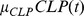	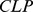 death
11	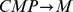	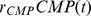	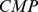 differentiation
12	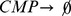	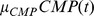	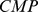 death
13	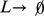	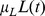	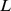 death
14	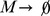	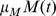	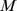 death

The time-dependence of the state variables has been explicitly stated in the transition propensities to differentiate the state variables from parameters.

The model also incorporates four different feedbacks from the blood cells 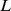 and 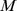 on to the 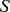 and 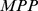 cells. Three of these, 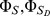 and 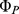, take the form
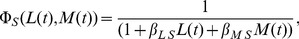
(1)where their respective parameters 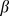 are defined in Table 2. These inhibit the activity of 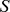 and 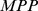 when blood cell levels are high. Specifically, 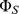 inhibits all 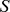 activity (both self-renewal and differentiation), 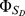 inhibits 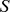 symmetric differentiation only and 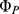 inhibits all 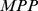 activity. The form of Eq. (1) is based on earlier studies [28], [38], and conforms to the assumptions that: 1) numbers of both blood cell types have an effect on 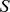 and 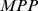 activity, 2) their effects are additive, 3) the strength is different for 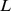 and 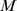 cells, and 4) when numbers of either fall, the activity of 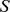 and 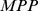 increases again. Note that feedbacks 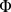 always take values on 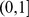.

**Table 2:** Constants and parameters in the stochastic model.

Parameter	Value	Description
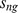	varied	Niche group size
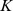	10	Niche carrying capacity of stem cells
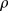	Eq. (2)	MPCR
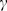	varied	MPCR parameter (exponent)
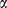	varied	MPCR parameter (multiplier)
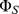	Eq. (1)	Feedback from 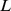 , 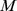 on 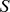 activity
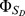	Eq. (1)	Feedback from 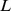 , 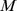 on 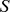 differentiation
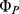	Eq. (1)	Feedback from 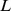 , 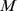 on 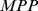 activity
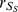	2.5	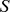 symmetric division (self-renewal) rate
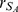	1	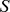 asymmetric division (self-renewal) rate
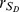	0.001	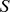 (symmetric) differentiation rate
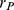	0.1	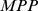 differentiation rate
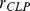	0.1	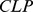 differentiation rate
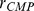	0.1	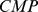 differentiation rate
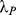	0.25	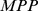 multiplication rate
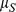	0.004	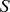 death rate
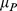	0.02	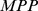 death rate
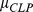	0.001	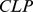 death rate
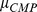	0.001	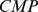 death rate
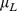	0.028	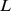 death rate
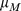	0.01	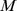 death rate
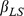	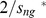	Feedback parameter of 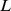 in 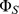
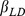	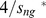	Feedback parameter of 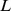 in 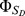
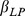	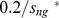	Feedback parameter of 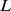 in 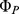
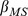	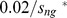	Feedback parameter of 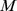 in 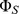
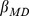	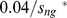	Feedback parameter of 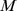 in 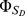
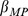	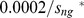	Feedback parameter of 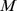 in 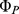

*Note: these parameters change depending on the niche group size, in order to maintain the same stable state at homeostasis, thus allowing equal comparison between them.

The last feedback is perhaps the most interesting, and is one aspect that differentiates this model from previous work. We refer to it as the Multipotent Progenitor Commitment Response, or MPCR [27]. This feedback determines the probability of an 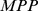 cell differentiating into either the lymphoid or myeloid routes. The idea behind this is that when blood cell numbers are not at their homeostatic levels (defined as a specific target value of 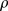), the MPCR aims to shift the production of new blood cells to the appropriate type. We model the MPCR as
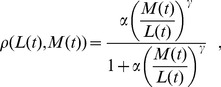
(2)where 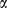 and 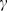 are positive parameters. When either 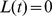 or 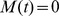 (states that are not reached in practice by the deterministic model, but do occur in the stochastic model) this causes a problem in Eq. (2), so in this event we simply treat 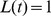 or 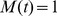, respectively, for the purposes of evaluating 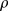; this has the advantage of affecting the value of 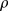 by only a small amount whilst keeping the MPCR pressure towards the correct cell type.

We set the MPCR parameters 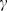 and 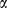 to give a target homeostatic blood cell ratio, which here is 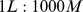 to loosely correspond to that in humans. To do this, we note that 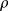 is defined as the probability of an 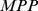 differentiating to a 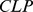, i.e. at homeostasis we have on average 
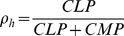
. From this, we can also specify steady states using the blood cell numbers, i.e. as 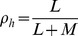, provided that the differentiation and death rates are identical for both 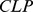 and 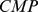, as well as 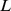 and 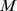 (however, we examine the general case and use a parameter setup where the death rates of 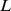 and 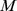 are not equal, but the only consequence is that the homeostatic state will not be exactly equal to 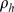 for the chosen 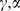; we explain this issue further in Supporting Text S1, Section 2). Now, at homeostasis we have 
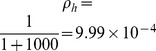
. We then substitute these values into Eq. (2), choose a value for 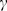 and so calculate the corresponding 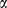. We can do this for different combinations of 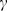 and 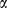, thus varying the strength of the response whilst retaining the same target cell ratio 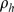.

Although many combinations of 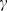 and 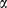 can give the same homeostatic ratio of 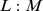, they strongly affect the sensitivity of the MPCR to changes in cell numbers and its response to perturbations. In [27], we used this model to examine the behaviour of the haematopoietic system from an evolutionary perspective. Treating it as a demand control system, where the demand comes from the entire organism, we showed that there is varying selection on organisms with different MPCR parameters 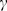 and 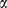. Different organisms can thus evolve a range of parameters as their environments vary, and this affects the dynamics of their haematopoietic system as well as its response to perturbations. This implies that it is important to take into account the evolutionary background of an organism when examining the dynamics of the haematopoietic system, and stem cell systems in general. This is consistent with the idea that stem cells are units of evolution [43], [44].

### Stochastic HSC Model

The system of ODEs for the deterministic HSC model (Supporting Text S1, Section 1 and Ref. [27]) can be considered the *continuously-conditioned average* of the stochastic system [45]. If these ODEs were linear, we could say that they represent the mean of the stochastic system (that is, the initially-conditioned average: see [45]); however, as they are non-linear due to the feedback functions, we cannot tell *a priori* the relationship between the deterministic and stochastic solutions (although having said this, initial explorations of a much simpler stem cell system found the ODE solution to be reasonably close to the stochastic mean in the case of a single lineage with feedbacks [15]). In general, ODE models are not able to account for the full range of dynamics of highly stochastic systems, and in extreme cases can even give results that are unrepresentative of the full behaviour of the system [46], [47]. The stochastic formulation of the ODE model also has six states and fourteen transitions between the states. However, rather than occurring at deterministic *rates*, these transitions now occur with particular *propensities* at each step of the simulation.

The stochastic simulation algorithm (SSA), developed by Gillespie [48], allows us to simulate such a system in a statistically exact way. We first describe it in general terms and then discuss its application to the HSC system. In general, we consider a set of 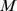 types of transitions between 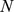 kinds of cells. We track cell populations through time with the state vector 
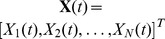
, where 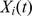 represents the number of cells of type 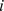 at time 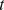 and 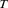 denotes the matrix transpose. We let 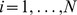 denote the cell type index and 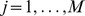 denote the transition index; boldface font represents a vector of size 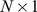.

The SSA is a simple and powerful method, and essentially consists of finding, at each step, the time until the next transition and which transition occurs. To do this, we define the 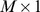 vector of propensity functions 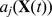, where 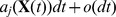 is the probability of transition 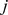 occurring in an infinitesimal time 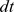, and where 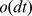 represents terms of higher order in 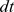 (for further details about the importance of this term, see [49]). In addition, we have a stoichiometric matrix 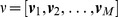 of size 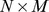, which represents how each transition affects the numbers of cells. Knowledge of 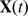,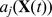 and 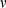 is all that we need in order to simulate the time dependence of the HSC system.

The time until the next transition, 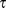, is sampled from an exponential random variable with parameter 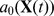, where
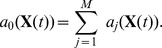



This implies that the probability of no transition in the next 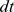 is 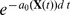, which can be expanded as a Taylor series to 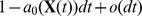. Given that a transition occurs, the probability that it has index 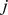 is
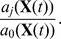



Once these two have been chosen, the state vector is updated as
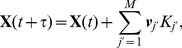
(3)where 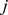 is the index of the transition that occurred and



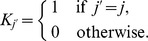



The SSA was initially developed to simulate the interactions of different chemical species in a dilute gas, and has since been extended to dilute solutions [50]. Both of these scenarios assume that the system is macroscopically well-stirred and homogeneous. The usual mass-action form of its propensity functions are directly based on these assumptions. In order to use the SSA with the HSC system, which does not necessarily obey either assumption, we adopt instead a *phenomenological* approach to definining the propensity functions, as is the custom when constructing ODE population models. In effect, we simply convert the transition rates of the ODE system into transition propensities. The form of the propensities depends on our assumptions regarding the processes involved: thus here, the propensities are dependent upon a rate constant, the population of the transitioning cell type, and in the case of stem and progenitor cells, also the feedbacks that we have assumed exist (Table 1). Note that the propensities give the probability of a reaction occurring *per unit time*, and therefore are not required to remain on 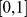. For our HSC model simulations, we define the state vector as 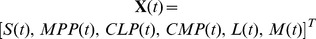.

### Fast Stochastic Simulations

The SSA framework of the previous section is both simple and statistically exact, meaning that a histogram built up of an infinite number of simulations is identical to the true histogram of the system. However, especially for systems with larger populations (generally, hundreds or thousands of cells, or more), faster transitions or those whose transition rates have a complicated form, it can become slow. For such systems, if computational time is an issue, it is more appropriate to use an approximate method. A common example of such a method is the 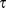-leap method [51], which evaluates many transitions in one (larger) step, thereby speeding up computation.

The 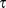-leap update formula also takes the form in Eq. (3), but rather than a single transition, now the number of transitions occurring in each channel 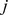 over each step 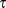, represented by 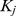, is given by
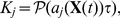
(4)i.e. it is a Poisson random number with mean 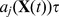. This approach can greatly speed up computation, although it incurs a loss in accuracy. The stepsize can be varied, and is commonly chosen to be sufficiently small to achieve reasonable accuracy but sufficiently large to increase the computational speed. A simple way of doing this is to bound the change in each cell population over one step, 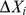, by a small fraction 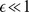 of 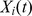. Since 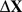 is a random variable, in practice this means bounding its mean and standard deviation. 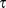 can then be chosen to be consistent with these bounds. For the simulations in this paper, we have used a simple version of this scheme (set out in detail in [52], specifically, Eqs.(32) and (33)), without any consideration of reaction criticality. Several similar methods have been proposed with higher efficiency or accuracy (for example, [53]–[55]). Since we introduce additional complexity by simulating an entire metapopulation of lineages and coupling them, here we have chosen to use a simple stepsize-adapting scheme.

### Simulating a Metapopulation of Niche Lineages: Vectorised 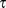-Leap

In order to simulate a large number of niche lineages, we expand the Gillespie SSA/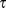-leap approach from just one sub-simulation (i.e., lineage) to many. By including interaction terms between each individual niche lineage, we can easily simulate an entire interacting heterogeneous metapopulation of niche lineages. The heterogeneity results only from intrinsic noise, that is, noise arising from random thermal fluctuations, which is present even in genetically identical populations in the same environment [35]. Our method almost resembles a compartment-based model, which consists of many discrete spatial compartments, each of which is assumed to be homogeneous inside. However, as details of the spatial aspects of stem cell niches are still emerging, we chose not to explicitly equate each sub-simulation with a discrete spatial compartment; rather, each sub-simulation represents a niche lineage whose physical locations are not taken into account.

We take advantage of the native matrix structures of the Matlab programming language, with the state vector of each niche lineage forming one column of the overall state matrix. Thus, if there are 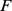 separate niche lineages, instead of an 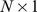 state vector, we now manipulate an 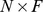 *state matrix*. This approach is conceptually simple, easily allows for the introduction of coupling and interactions, and is especially fast (as Matlab is optimised for matrix calculations, calculating each step of the SSA scheme on a matrix rather than a vector has little effect on the speed, whereas doing the same for each niche lineage in turn would be very much slower). This state matrix approach could easily be implemented in other programming languages, and although it would not necessarily result in a large computational speedup (for instance, this is likely to be the case in the popular programming language C), we argue that it is favourable even for its inherent simplicity alone.

Since each sub-simulation of the SSA chooses timesteps randomly, the metapopulation of niche lineages would not be simulated in time synchronously, akin to a running race where some runners are ahead and some lag behind. Since we want to simulate an interacting, coupled metapopulation, all lineages must stay in step otherwise the interactions would effectively be averaging over time. The solution is to switch to the 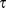-leap method from the previous section, use it to choose a suitable timestep and evolve every niche lineage over this timestep. It is important to note that this does *not* bias our results in any way: we are only selecting a common timestep for all the lineages, but the reactions that occur in each lineage are then chosen according to the true Markov process.

To explain this, let us go back to basics: the evolution of each lineage is governed by a Markov jump process [56], which is approximated by the 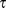-leap method. If we wanted to simulate a population of 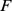 niche lineages using a standard 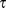-leap, we would run 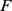 repeat simulations of a single lineage. This could be done with either a fixed or an adaptive timestep, and we would sample the Markov process (carry out the 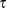-leap update) at the time points given by those timesteps. However, the process itself is *independent* of the times at which we sample it (although, of course, the same cannot be said for the solution of our approximate 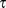-leap method, which approaches the true Markov process as the timesteps decrease). Thus we are free to sample the Markov process at whatever time points we choose, provided we remember the condition on our approximate solution. Now, a reasonable part of the computational time of a leaping method is taken up with the overhead of calculating the timestep adaptively. By simulating the metapopulation simultaneously, our method allows us to choose just one timestep for all 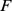 niche lineages, reducing the total overhead. The only disadvantage is that if one lineage contains unusually large populations, this would pose as a bottleneck on the common stepsize.

We must thus find the common limiting timestep from the whole metapopulation. First, the propensities of each transition in each niche lineage are calculated. Then, we find the lineage with the largest 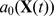, that is the sum of the propensities. Now, we simply continue with the stepsize selection as if we were only simulating a single lineage, and its propensities were those of the selected one. Once the stepsize has been chosen, the entire metapopulation is evolved over that step using Eqs. (3) and (4). We describe this more precisely in Algorithm 1.

#### Algorithm 1

Vectorised 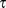-leap

*At time* 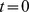 *, with a metapopulation of niche lineages of size* 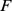 *, each taking initial states of* 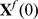, 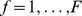:

0. Initialise state matrix containing 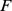 niche lineages, each with 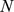 distinct cell types: this is an 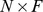 matrix containing the initial state vectors 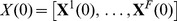. *With the system in state* 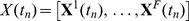 *at time* 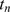:

1. Calculate propensities of each niche lineage to get an 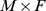 matrix of propensities, 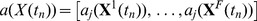, 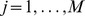.
2. Find 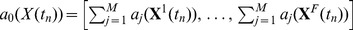.
3. Find 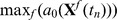, 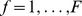, the niche lineage with highest total propensity, and assign its lineage index to 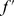.
4. Calculate 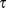 using the stepsize-adapting procedure in [52], with the propensities 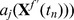, 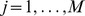.
5. Update state matrix as 
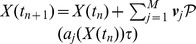
, and 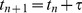. If any cell type in any niche lineage goes negative, redo step using 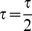. Otherwise, return to Step 1.

We select the lineage index of the highest total propensity, as this is the niche lineage with the most frequent transitions, and thus the limiting factor on the stepsize. Of course, the actual number of transitions at each step is probabilistic, so if by chance too many transitions occur for any cell type in any niche and its population goes negative, the step should be redone with 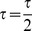 (standard procedure in 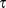-leap methods). For even tighter control of the stepsize, instead of selecting a single niche lineage 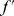 and taking its total propensity as the limiting factor, we could instead find the lineage index of the maximum propensity of *each* transition. This would set a tighter bound on 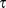, as each transition would partake in the stepsize-selection process. However we found the current method to be satisfactory.

 Although in this paper we have used a procedure from Ref. [52] to find the timestep, we are not restricted to this particular method. The matrix scheme we have described above is flexible, in that it can easily be fitted into *any* procedure for adapting 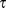, including advanced and efficient methods such as the Stochastic Bulirsch-Stoer method [55] or the Theta-trapezoidal 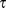-leap method [53]. As long as we find the niche lineage with the most frequent reactions, we can choose a timestep based on this lineage for the entire metapopulation using any 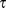-adapting scheme.

### Coupling Niche Lineages

Each HSC niche does not exist in isolation in the bone marrow; in fact HSCs often circulate around the bone marrow and bloodstream [57], [58]. Differentiated blood cells are also, in general, ejected from the niche and enter the bloodstream, although certain differentiated cell types can remain localised to the niche [12]. Thus, cells from each niche lineage are mixed to various degrees after they have fully differentiated and leave the niche. To investigate the dynamics of coupling together separate niche lineages, we introduce the implementation of the coupling.

We assume that there is no interaction between cells that are not fully differentiated (that is, any cell type except for 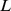 and 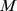). The coupling comes into effect only through the feedback functions of the 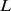 and 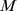 cells on to 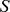 and 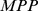 cells (although it should be noted that our computational method can handle any form of coupling). To capture this, we create ‘niche groups’, where the feedbacks on the stem and progenitor cells in each niche lineage depend on the *total* levels of 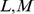 in the entire niche group of that lineage. In practice, this means that the blood cells 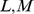 in each lineage of a niche group are replaced in the feedback equations by the total 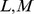 in that niche group (whilst normalising the parameters by the niche group size). The propensities for each niche lineage are then calculated as described in the previous section and the populations of each niche lineage updated separately (Algorithm 2).

**Figure 2:**
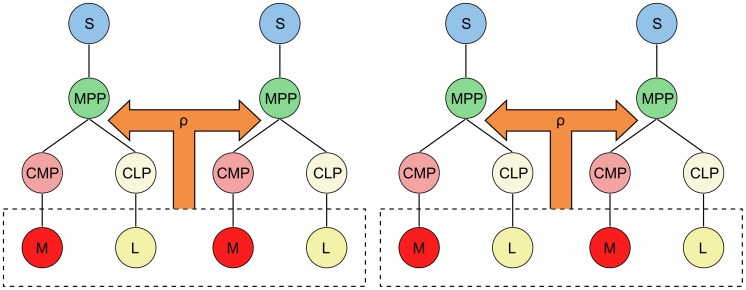
A population of four coupled niche lineages with a niche group size of two. The MPCR from the total 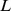 and 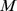 cells in the niche group is fed back to both lineages. This is also the case for the feedbacks 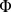, which are not shown.

To aid in visualising this, we give an example using a population of four niche lineages coupled into niche groups of size two, i.e. 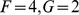 (Fig. 2). When the lineages are coupled, the feedbacks are taken over the total 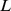, 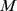 in the respective niche group. Then, denoting by 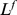 the population of 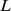 from niche lineage 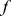, and similarly for 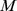, the feedbacks of the first two niche lineages would be 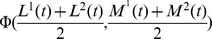, and the last two would be 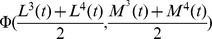. This is the case for all feedback functions, including the MPCR. The factor of one half is necessary to normalise the steady states to be directly comparable, regardless of niche group size.

#### Algorithm 2

Coupled vectorised 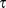-leap

*With the system in state* 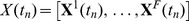 *at time* 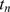 *, and* 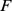 *niche lineages coupled into* 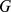 *niche groups, i.e. niche group size* 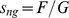:

1. Find total 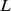, 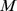 for each niche group, 
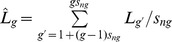
, 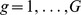; i.e. take the sum of all 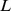 over each niche group and normalise by niche group size, and similarly for 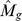.
2. Calculate MPCR values 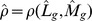, 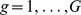, and similarly for feedbacks 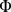 to find 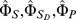. This gives a vector with length 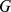 of values for each feedback function.
3. From these, formulate individual feedback functions for each niche lineage (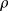, 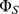, 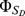 and 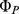) by taking 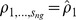, 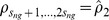,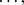 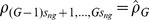, and similarly for 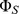, 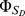 and 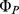 (i.e. assign to each individual niche lineage's feedbacks the value of its niche group's feedbacks). These are vectors of length 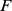.
4. Now proceed with Steps 1 to 5 of Algorithm 1.

This method allows us to evolve an entire metapopulation of niche lineages in time, and to take into account the interactions between the blood cells of different lineages in the feedbacks.

## Results

### Fast Stochastic Simulations

We begin by evaluating the performance of our computational method. Although it is not exact, the 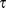-leap is in general a much faster simulation method than the SSA. The error parameter 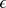 (introduced in the Fast Stochastic Simulation section) indicates the amount of error we allow into the leaping approximation. Common values for 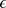 are of the order of 0.01, meaning roughly that the timestep selected allows at most a 1% change in the population of the rarest cell type; a value of 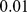 typically corresponds to high accuracy and 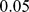 to low accuracy, but this can vary.

We ran simulations of a metapopulation of 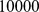 uncoupled niche lineages with the vectorised 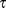-leap method described in Algorithm 1 for a wide range of values of 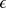, as well as with a vectorised SSA, and recorded the average runtimes on a standard desktop computer. The SSA can be regarded as finding the exact solution (for uncoupled niche lineages only — it loses this exactness when the lineages are coupled, see Vectorised 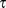-leap section). Therefore we compared the probability density functions (PDFs) returned by the 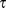-leap to the exact PDF given by the SSA to get an idea of how the errors of the 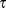-leap simulations changed as the error parameter was varied.

The simulation runtimes are listed in Table 3, as are the total errors of the 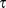-leap results. We calculated these by taking the 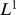-distance between the weight of each bin (that is, probability density multiplied by bin width) of the 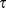-leap PDFs and that of the SSA. The runtimes decrease as the error parameters increase, with the SSA taking the longest, as expected. The self-distance of two different SSA simulations is relatively large (Table 3, top row), indicating that the differences in errors between the 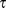-leap with 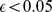 may be due to Monte Carlo error. This means that the vectorised 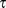-leap with these error parameters is about as accurate as the SSA. With 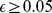, however, the 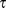-leap does become substantially less accurate. Accordingly, in the rest of our simulations, we used 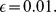 Table 3 shows that the vectorised 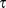-leap is indeed faster than the SSA, significantly so when 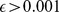. However, even with 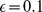, the 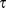-leap finds remarkably accurate solutions. This is compounded with the fact that the SSA should not be used to simulate coupled niche lineages, as each lineage proceeds at its own pace. These factors mean that approximate, fast methods that can sample the state matrix synchronously are most ideal for simulating larger, interacting systems such as our HSC system.

**Table 3:** Runtimes and errors of the vectorised 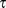-leap method compared to the SSA.

Simulation method	Runtime (hours)	Total error
SSA	67.4	0.201
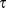 -leap, 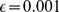	44.6	0.173
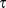 -leap, 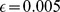	6.7	0.175
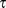 -leap, 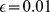	2.9	0.189
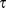 -leap, 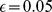	0.9	0.214
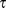 -leap, 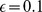	0.7	0.312

The errors are calculated by subtracting the weight of each point of the PDF (that is, value multiplied by bin width) from the corresponding point of the SSA PDF. The error in the SSA row is the SSA self-distance, i.e. the error between two different SSA simulations. These simulations are of uncoupled niche lineages *only*, hence the SSA can be regarded as the true solution.

### Stochastic Model Dynamics

We then ran simulations of the HSC system on metapopulations of 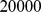 uncoupled and 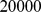 coupled niche lineages for each set of parameters, using our vectorised 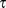-leap method from above with 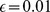. In order to investigate the coupling between different lineages, this was grouped into sub-populations (for example, 200 sub-populations of niche groups of size 100). The model is not parametrised using any specific data: the parameters in Table 2 are a canonical parameter set, chosen to elucidate general principles rather than make specific biological predictions. Due to the number of parameters, a thorough parameter sweep or sensitivity analysis was beyond the scope of this paper; however, manual experimentation using several parameter sets showed relative robustness in the system dynamics (for instance, see Supporting Text S1, Section 3). In one or two cases, we observed consistent oscillations in cell populations, qualitatively similar to Ref. [59]; here, we have used parameters that settle down to homeostatic cell populations. Between 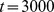 and 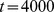 seconds, transitions do not occur faster, as it may seem from some of the plots; not all transitions are recorded, and we have sampled the ones in this time period more often to give an accurate picture of the system dynamics after a perturbation.

**Figure 3:**
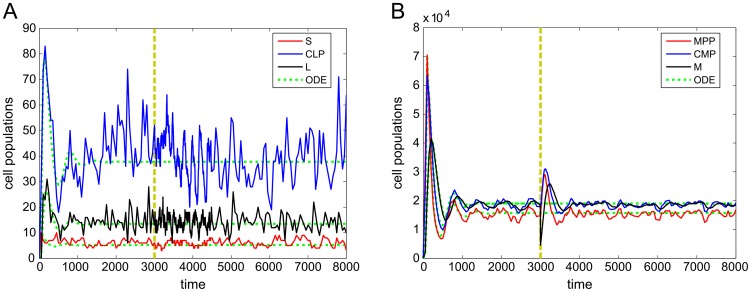
Single stochastic trajectories of all cell types over time. Shown are levels of A) 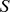, 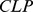, 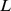, and B) 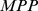, 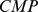, 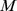 in a single niche lineage over the full simulation time. For comparison, ODE trajectories (with no perturbation) have been included. Yellow dashes show time at which the lineage is perturbed by removing 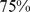 of its 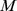 cells.

We elucidate the basic dynamics of the model in Fig. 3, which shows a stochastic simulation of a single niche lineage along with the ODE model for comparison. We started all our simulations in the state 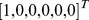, i.e. with one 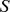 and no other cells. All cell populations experience an initial surge, which then dies down to a steady state. At 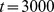 seconds, we perturbed the 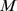 cells by removing 75% of them (indicated by yellow dashed line; ODE model not perturbed). The 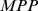 and 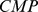 surge just after the 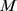 are depleted, but there seems to be little response from the 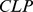 and 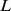 cells. Significantly, there is also little response from 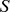 cells. After around 1000 seconds the 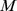 cells return to their pre-perturbation numbers, and all three cell types then settle back to their steady states. We set the MPCR parameters to reach homeostasis at the ratio 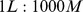 (corresponding to 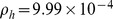). However, as the death rates of 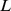 and 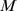 were not equal, we did not expect to observe this exact homeostatic ratio; indeed, Fig. 3 shows that the homeostatic state of the model using this particular parameter space is around 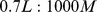, corresponding to 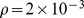 from Eq. (2) (see HSC Model section and Supporting Text S1, Section 2). The ODE model roughly follows the stochastic simulations, with both indicating similar homeostatic states.

**Figure 4:**
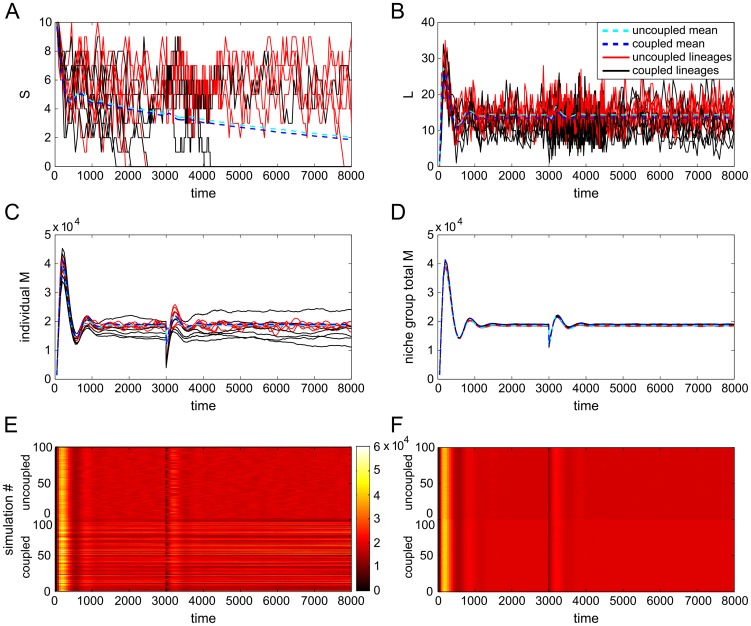
Trajectories of stochastic simulations of uncoupled and coupled niche lineages. Shown are six individual lineage A) 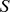, B) 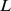 and C) 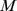 cell levels over time, with means superimposed; D) total 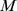 (normalised by niche group size) for six uncoupled and six coupled entire niche groups (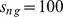) over time; E) trajectories of 100 simulations of uncoupled (top half) and coupled (bottom half), where colour represents the populations of 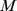 in each lineage, and similarly for F), where colour now represents total niche group 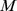, normalised by niche group size.

In Fig. 4A,B,C we show the time evolution of six separate simulations each, of both uncoupled and coupled (niche group size 100) niche lineages. The first thing we notice is that the 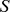 cells in some lineages die out (Figs. 4A and S1), but the rest of the lineage keeps functioning (Fig. S1). Over one quarter of all lineages had lost their 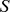 by 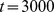 seconds, and this number went up to over one half by the end of the simulations. Only in a handful of these cases did the entire lineage die out; the rest were maintained by the 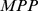 cells. Next, the total 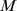 numbers per niche group (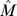, normalised by niche group size; Fig. 4D) are close but not identical for uncoupled and coupled niche lineages. This is supported by Fig. 4F, where colour indicates 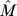 numbers and which shows 100 trajectories each of uncoupled and coupled niche groups. The 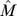 numbers are consistent for all niche groups, and there is also little difference between uncoupled and coupled 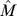 numbers. In contrast, Fig. 4E highlights the differences between 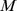 per individual lineage seen in Fig. 4C: uncoupled lineage 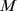 numbers fluctuate in an uncorrelated way over time and all lineages behave in a similar way, whereas those of coupled lineages show a distinct correlation over their own trajectories, as well as considerable variation between individual niche lineages. Fig. S1 demonstrates that this also happens, to varying degrees, for the other cell types. It is difficult to tell whether this is also the case for 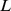, where stochastic fluctuations are large compared to cell numbers, but Fig. S2 helps to clarify the issue: the steady states of the uncoupled and coupled 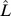 are also fairly close but not identical (Fig. S2A,C), and in Fig. S2B we can make out the distinct lines made by the coupled lineage 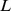 levels, implying their fluctuations are correlated compared to the uncoupled lineages. To sum up so far, Figs. 4, S1 and S2 tell us that 1) although there is a large surge in 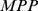 numbers, there is a smaller relative response in numbers of 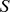; 2) there is also a large surge in 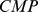 numbers to replenish the lost 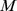, which corresponds to a modest drop in 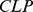 and 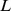 numbers followed by a small surge to return to their steady states; 3) cell populations in individual uncoupled niche lineages fluctuate considerably with time, whereas those of coupled niche lineages less so; 4) however, cell numbers between individual coupled lineages are much more varied than those of uncoupled lineages, which are all roughly similar.

### HSC Steady State Distributions

#### Varying MPCR parameters

In [27], we investigated the dynamics of MPCRs with different parameters 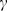 and 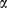 and showed that different values give a different response following a perturbation; thus they are linked to the evolutionary background of the organism. In this paper, their values were always chosen to give 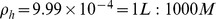, to approximately correspond to the ratio of blood cells in humans. As the choice of values is constrained to the curve given by 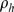, we henceforth refer only to 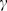, with the implication that 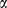 is also varied according to this curve. 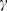 can take on any positive value; zero implies a non-responsive MPCR, that is it does not react to changes in 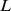, 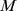; as 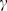 increases, so does the strength of the response to non-homeostatic ratios of 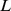, 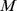. Once 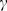 goes into the tens, the MPCR is extremely reactive, even creating extra fast-scale fluctuations in the post-perturbation cell numbers on top of the normal fluctuations involved in relaxing back to homeostatic levels. Above this, it becomes impossible to evaluate in practice, as 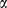 is too small. Therefore, reasonable values for 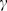 most likely lie somewhere in the range from 0.1 to 5.

**Figure 5:**
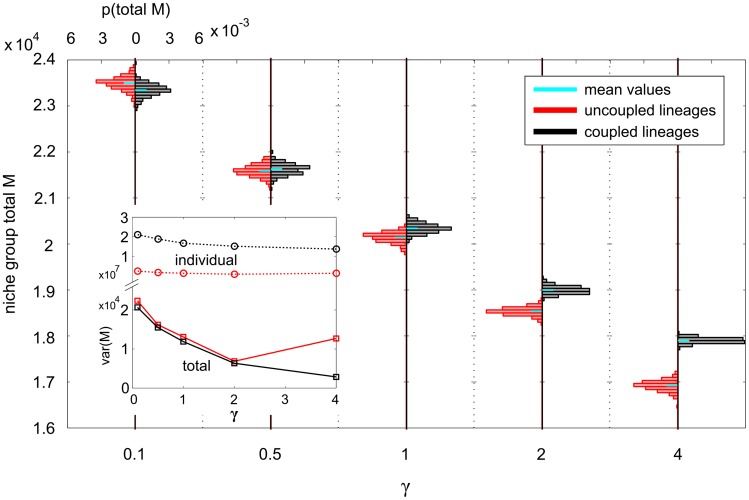
PDFs of both uncoupled and coupled total niche group *M*, for five different MPCR parameter sets. The parameters 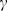 and 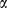 were always set to give cell steady state ratios of 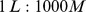. The plot consists of ten PDFs, five each of uncoupled and coupled niche lineages. The axes for each PDF are identical, and quantified on the left and top. MPCR parameters are varied on the bottom axis. The inset shows the variance of each PDF as a function of 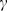 (note the broken y-axis).

Now, we examine the distribution of each cell type at homeostasis and how the choice of 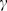 and 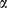 affects the steady-state behaviour of the HSC system. As 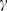 is increased, so the mean values of the cell distributions change. For some cell types the means increase (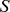, 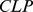, 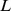), and for others they decrease (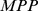, 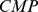, 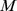), following the dynamics of the ODE model. Associated with these changes in the mean are corresponding changes in the variance of the distribution of each cell type: increasing mean also implies increasing variance, and decreasing mean decreasing variance. As examples, we highlight 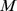 (Fig. 5), 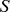 (Fig. S3) and 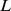 cells (Fig. S4), and summarise for all cell types in Fig. S5.

**Figure 6:**
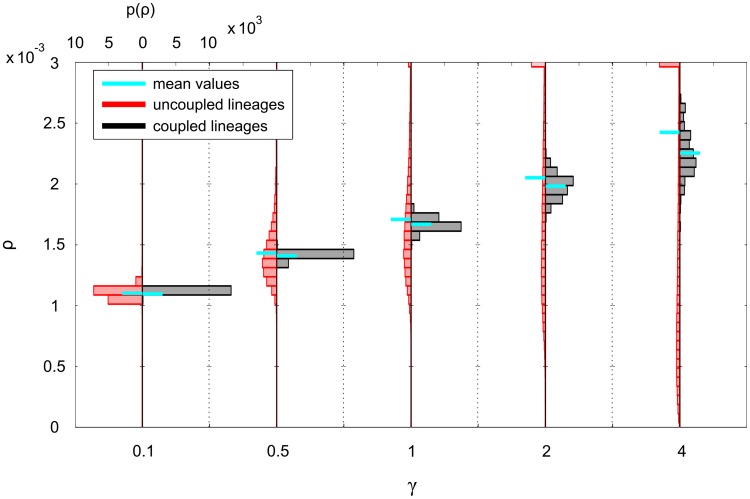
PDFs of both uncoupled and coupled MPCR values in each individual niche lineage, for five different MPCR parameter sets. The axes for each histogram are identical, and quantified on the left and top. MPCR parameters are varied on the bottom axis.

The distribution mean of the MPCR also increases with increasing 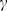, as does its variance (Fig. 6). Although the mean MPCR remains reasonably close for both coupled and uncoupled lineages, the uncoupled MPCRs have a particularly high variance, with the bulk of the distribution away from the mean as well as a long tail. The mean values of the 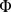 feedbacks also increase with 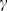 (very little in the case of 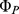; Fig. S6) but their variance does not seem to change consistently. However, it is possible that we observed this because the variances are very low (between 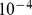 and 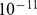). The 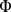 feedbacks take values consistent with the 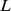, 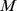 cell populations.

Thus different 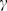 (and 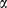) parameters change the MPCR dynamics, which affects the homeostatic cell populations, which then affects all four feedbacks, which in turn affects the cell populations, and so on. We find that both coupled and uncoupled niche lineages behave in a similar way as the MPCR parameters are altered, albeit to varying degrees. We explore more fully why the cell populations are affected by MPCR parameters in Supporting Text S1, Section 2.

#### Coupling niche lineages

**Figure 7:**
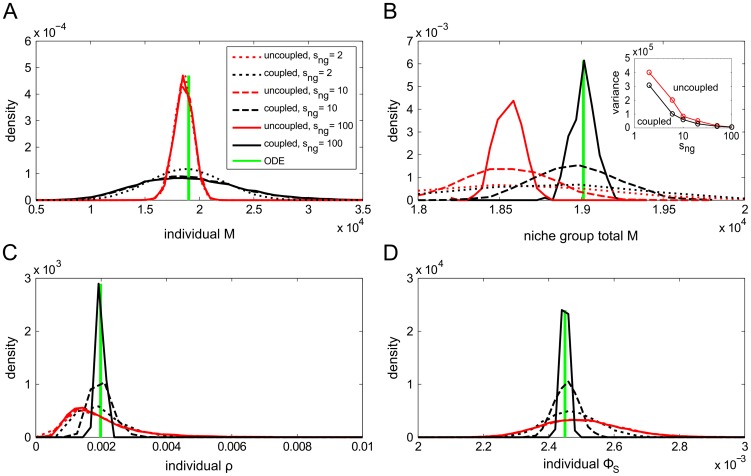
Steady-state PDFs of *M* cell levels and MPCR and 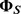 feedbacks for various niche group sizes. Shown are A) individual niche lineage 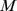; B) total niche group 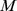 normalised by niche group size (inset shows the variance of the PDFs as niche group size is changed); C) individual niche MPCR values; D) individual niche 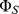 at steady state, i.e. 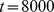 seconds.

We now fix the MPCR parameters at 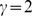 and 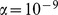, to again correspond to 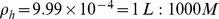. These values represent a reactive but not hyperactive MPCR intended to highlight any dynamics arising from coupling niche lineages, to which we now turn our attention. When taken individually, it is the uncoupled niche lineages that are regulated more tightly, with the 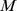 numbers of the coupled lineages having a much wider distribution (Fig. 7A). In contrast, from a systemic view the situation is the opposite: when looking at total cell numbers per niche group (normalised by niche group size), the coupled niche groups 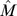 have narrower distributions compared to the uncoupled ones (Figs. 7B and S5). This comes about because when niche lineages are coupled, blood cell numbers are regulated only at the niche group level, allowing the blood cell numbers in individual lineages to vary widely.

A key difference between the distributions of the coupled and uncoupled niche group cell numbers is their mean (Figs. 7A,B, S7A,B and S5). Of course, this is also true for individual lineage cell populations, but is harder to notice visually; when the cell numbers are summed over niche groups, the distributions of the coupled and uncoupled niche lineages are separated (Figs. 7B and S7B). In all cases, the coupled and uncoupled lineage cell numbers are centred around different values. However, as the MPCR parameters affect cell steady state populations, it is not trivial to pin down which distribution is more closely centred around the target cell ratio 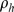. Using a different model parameter setup (with equal death rates, thus allowing the system to reach exactly 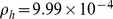), we found that it was indeed the coupled niche lineages that regulated their cell populations to be closer to 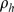 (Supporting Text S1, Section 3).

The corresponding homeostatic distributions of two of the feedback functions are shown in Fig. 7C,D. In contrast to the cell populations, it is the feedbacks of coupled individual niche lineages that are more tightly distributed, and this effect becomes stronger as niche group size is increased. This suggests that it may be due to the niche lineage grouping, because within each niche group the feedbacks are identical. To check this, we next calculated the mean feedbacks in each niche group. It turns out that the distribution of the feedbacks is indeed controlled by the coupling, and the mean feedbacks per niche group have similar distributions, whether they are coupled or uncoupled (Fig. S8). The figure also shows that the niche group size changes the feedbacks' distribution means. This is again a case of the coupled MPCRs affecting the mean cell numbers in each niche group, which then affect the 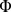 feedbacks, which in turn affect the cell numbers.

We find that coupling individual niche lineages together into niche groups, by pooling the blood cells of the group in the feedbacks, has an effect on the distributions of the cells as well as of the feedbacks. This effect is positive, in that it allows the blood cell numbers to be regulated more closely to the target homeostatic levels dictated by the model.

### Perturbation Analysis

Next, we look more closely at the response of the system to perturbations. We examine three types of perturbation: even perturbations (37.5% reduction of 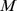 from every niche lineage), uneven perturbations (75% reduction of 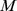 from every *second* lineage only), and random, or more precisely, probabilistic, where each lineage has a 50% chance that its 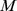 are reduced by 75%. The perturbations were chosen to cause, on average, an identical change in cell numbers across the entire population of niche lineages, that is the removal of 37.5% of the entire population of 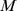. The actual values of 37.5% and 75% are illustrative in nature, rather than realistic examples of blood loss from injury.

The response of the system to perturbations is given by two main indicators: return time to homeostatic levels, and overshoot/oscillation size, defined as the difference between the maximum of the post-perturbation spike in cell numbers (and feedbacks) and their steady states. Return time, much like the homeostatic levels of the system, is dictated by the model parameters. Moreover, it is difficult to accurately measure, as even in homeostasis, there is a continuous turnover of cells, leading to fluctuations in the cell numbers. We did not find a substantial difference in return time between uncoupled and coupled niche lineages for any type of perturbation, and the ODE model and the mean of the stochastic system closely matched in this respect.

#### Coupling niche lineages

**Figure 8:**
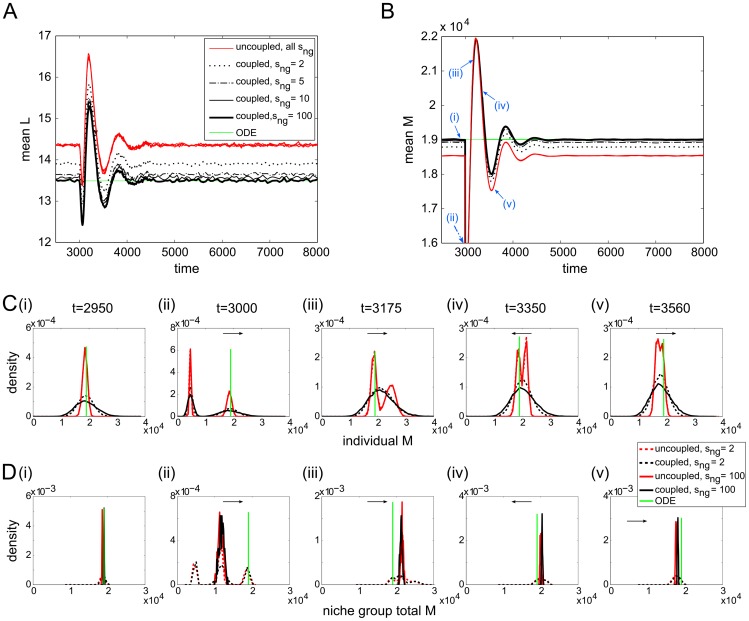
Evolution of population means and distributions of cell levels around the perturbation. Population means of A) 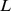; B) 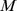 for various niche group sizes during and after the perturbation. In addition, we plot PDFs of C) individual lineage 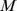 and D) total niche group 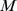 at the time points labelled with blue arrows in B). Arrows indicate which direction the peaks are moving with time.

In the interest of brevity, we first restrict ourselves to a random-type perturbation only and again fix 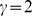 and 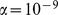, and focus on coupling niche lineages. We have already seen that the distribution means of both coupled 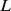 and 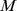 more closely approached the target 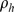 as niche group size was increased; this is supported by Fig. 8A,B, which show the mean 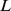 and 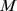 over time. It is important to realise that this is *not* a result of the averaging process to calculate total niche group 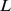 and 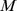. As a control, we also plot the distribution means of the uncoupled niche lineages, each of which were summed over niche groups as with their coupled counterparts; their mean numbers are so similar that they are almost indistinguishable from each other in the figures. In Supporting Text S1 (Section 3) we show that the ODE model does give a good indication of the target mean cell populations for a given parameter set; the mean 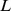 and 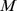 approach the ODE solution as niche group size is increased (Fig. 8A,B).

We examine the distributions of 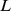 and 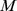 at various times throughout a random-type perturbation and its aftermath (Fig. 8C,D; the distribution peaks move in the directions specified by the arrows). We begin at 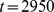 seconds, with the system in its homeostatic state. At 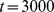 seconds, the perturbation is applied, reducing the 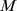 cells of roughly half the niche lineages by 75%. This results in a bimodal distribution of 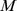 (from unperturbed and perturbed lineages) for both uncoupled and coupled niche lineages (Fig. 8C(ii)); when 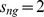 the distribution of total niche group 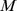 is trimodal, since the possibilities are either zero, one or two perturbed niches per niche group (Fig. 8D(ii)). By 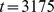 seconds, the individual coupled lineages' 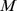 cells had resumed their previous unimodal shape, but the uncoupled niches retained their bimodality (Fig. 8C(iii)). By 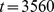 seconds, the individual uncoupled lineages' 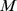 cells were also starting to coalesce into a unimodal distribution again (Fig. 8C(v)). Throughout, except for very close to the perturbation time, the distributions of the total niche group 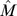 with 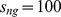 kept their shape, with the coupled lineages remaining centred closer to the target homeostatic state (Fig. 8D).

**Figure 9:**
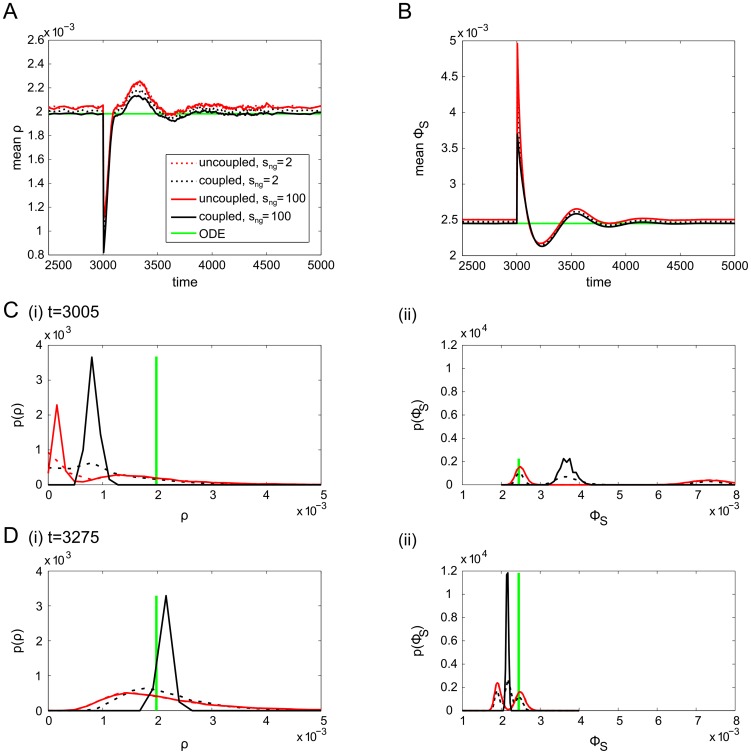
Evolution of population means and distributions of feedbacks around the perturbation. Population means of A) MPCR values and B) 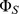 for various niche group sizes during and after the perturbation. In addition, C) shows PDFs of individual MPCR and 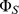 values at 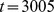, and D) at 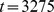 seconds.

Repeating this for the MPCR and 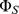 feedbacks, we see that the response of the feedbacks after the perturbation is approximately similar, albeit again with small differences in steady state (Fig. 9). Similarly to 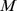, the uncoupled lineage 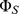 take a long time to recover, and even after over 200 seconds they have not returned to their initial unimodal distribution. In contrast, the coupled 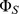 was already re-forming its unimodal distribution 5 seconds after the perturbation.

#### Different perturbation types

**Figure 10:**
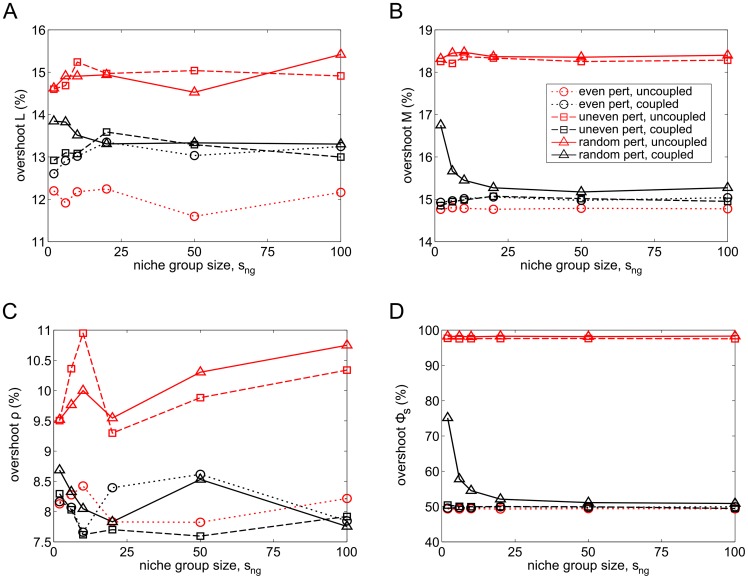
Overshoots of mean cell levels and feedbacks for various niche group sizes and perturbation types. Overshoots of mean A) 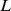; B) 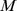; C) MPCR; D) 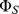 for various niche group sizes and three perturbation types. An even perturbation signifies a 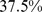 reduction of 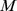 in every niche lineage, uneven means a 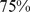 reduction of 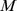 in every second lineage and random means a 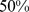 chance of each lineage losing 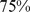 of its 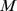.

Finally, we investigate how the overshoots of the mean cell and feedback levels vary for all three different perturbation types: even, uneven and random (Fig. 10). The overshoot response of the cell populations is different for each perturbation type (Fig. 10A,B): even perturbations affect all lineages equally, with the overshoots of uncoupled lineages slightly lower than coupled ones. Uneven perturbations, where the 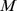 of every second niche lineage are perturbed, result in a smaller overshoot for coupled lineages than uncoupled ones, but this does not vary with niche grouping size. In contrast, random perturbations result in both a difference in overshoot between coupled and uncoupled lineages, with coupled ones having smaller overshoot, as well as a further decrease in the overshoot of the coupled lineages as more and more lineages are coupled together. The feedbacks also respond in a very similar way (Fig. 10C,D). Thus, we see that the response of the system is strongly dependent on perturbation type, with niche group size having no effect in the case of even and uneven perturbations, but random perturbations eliciting a more ideal response when the niche lineages are coupled in larger groups.

## Discussion

Most of the results above were concerned with linking together separate niche lineages into groups. A large niche group size indicates that the feedback from the blood cells (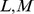) to the primitive cells (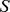, 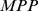) is regulated by a large fraction of the overall blood cell numbers in the organism. We found that as niche group size was increased, the mean levels of 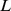, 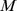 moved closer to the ODE model solutions. This is not a huge surprise: summing the blood cells in each niche group and normalising is equivalent to averaging over niche groups; the larger the niche group, therefore, the less the noise in total cell numbers per niche group, and the closer the system is to the ODE model. This is also a possible explanation for the lower variance of cell distributions in coupled niche groups. This reduction in noise can be useful for biological systems, for which noise is often detrimental. However, the question remained of whether it was the uncoupled lineages or the coupled ones (and the ODEs) that better achieved the target cell populations. From the control system perspective that we have taken, good control is defined as regulation of the cell populations to the target ratio 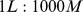. Given the interactions of the MPCR parameters and this ratio in setting the cell steady states (see Supporting Text S1, Section 3), it was the ODE solutions, and therefore the coupled niche lineages, that followed the target cell levels more closely than the uncoupled ones. Thus, it seems that on a systemic level, it is advantageous to connect together niche lineages. This hints at some intriguing possibilities for understanding the emergence of tissues, which are interacting populations of single cells.

The difference between the overshoots for the three perturbation types can be understood as follows. The even perturbation should result in a similar overshoot from both uncoupled and coupled niche lineages, since it affects all niches equally. This is roughly consistent with our results for 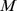, but it is unclear why the overshoot of the uncoupled 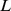 is considerably lower. The uneven perturbation affects uncoupled and coupled lineages differently, with coupled niches having smaller overshoot, but there is no variation with niche group size. Because it is a regular perturbation, coupling lineages (into even-sized groups) reduces the niche group overshoot, and it does not change with niche group size as in every case 37.5% of the cells in each niche group are lost. However, random perturbations elicited yet another response. With smaller groups or individual lineages, it is more likely that the entire niche group is perturbed, resulting in a larger overshoot. At the extreme ends of the scale, one could conceivably have one niche group with all niche lineages perturbed, and another with none. As niche group size is increased the chances of this decrease and the percentage of total niche group 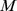 that is lost tends asymptotically to 37.5%, with the overshoot declining to the same levels as for an uneven perturbation. This shows that in environments with even perturbations, it may be advantageous to not couple niche lineages – however such environments are unlikely to occur in nature. In contrast, in natural environments with random perturbations, coupling niche lineages results in a more favourable response. This overshoot of blood cells following a perturbation is an important aspect of our model. There has been little work on this, although experimental studies have found that some types of T-cells are reconstituted very quickly and exceed normal levels, possibly supporting our results [60], [61]. We do not know of similar results for other blood cell types.

An interesting result from our simulations is the large variation we see in cell populations of coupled lineages between different lineages in the same niche group, and the relatively low variation over time of the populations in each lineage. This indicates that the activity of the primitive cells of each lineage varies, with some inactive/less active and others continuously differentiating to produce more cells, in order to achieve the correct homeostatic cell levels, somewhat akin to the HSC subsets found by Sieburg et al. [62]. Although we have not explicitly considered it here, our model also naturally captures the cycling behaviour of HSCs, with periods of quiescence and activity in each lineage [63]. In addition, after a perturbation, our model finds a response from both stem and progenitor cells. This is in agreement with studies finding stem cell activation after injury (e.g., [29]), but also supports the suggestion that at least part of the response is from progenitor cells [64].

Our results indicate that, in order to regulate blood cell populations tightly and for a less severe response following random perturbations, it is advantageous to the organism to couple haematopoietic lineages together via the feedbacks from blood cells on to primitive cells. There are three biologically-viable possibilities for the nature of this feedback mechanism: lineage-dependent feedback, where the primitive cells in one lineage can only sense numbers of their own differentiated progeny; local feedback, where the primitive cells can sense blood cells of any lineage in proximity to them; global feedback, where all primitive cells can sense all blood cells in the organism. Lineage-dependent feedback would require a biochemical mechanism in which niche lineages (or niche groups) can identify signals from their descendants and respond to the demand control from those cells, but not others in the blood; this could imply an epigenetic process. Indeed, studies have found that stem cell daughters of HSCs have a similar lifetime to their parents [34], and such an epigenetic mechanism could also exist in non-primitive progeny to regulate their feedback. Local feedback implies a spatial constraint on the feedbacks; although this has already been found to exist in the case of certain HSC progeny as well as other niche cells [12], it may not be a universal mechanism for the haematopoietic system because most blood cells enter the bloodstream rather than localising around the niche. However, in other stem cell systems, it is quite a plausible mode of feedback [65]. Finally, global feedback would require the HSCs to sense every blood cell in the bloodstream. Since it is likely that the feedbacks from the blood cells occur via growth factors [28], which naturally have a limit on their range of action, it does not seem likely that the HSC system incorporates global feedbacks from all blood cells. More likely is some combination of the above mechanisms. Looking for groups of epigenetic markers shared by HSCs, progenitor cells and differentiated blood cells could be a useful avenue for further experimental work. Finally, as evidenced by the dynamics of our model, the feedbacks are essential for achieving homeostatic cell rates [28]. Although we have not explored this issue further, our results also support the idea that cancers may be a failure of the signalling mechanism and the associated feedback control [66].

In ODE models, we can only account for a single, or at best an identical set of deterministic niche lineages, so that the interactions between a *heterogeneous metapopulation* of lineages is underexplored theoretically. This is important for two reasons: first, the dynamics of the entire system cannot be determined just by looking at its parts, and second, we can take a much broader point of view by looking at an entire population [26]. Indeed, Huang [32] suggests that this is one of three as-yet-neglected perspectives that should be adopted in stem cell modelling. For example, maintaining homeostasis at the population level can be achieved by several possible strategies [64]; only looking at a single stem cell restricts consideration to just one strategy, asymmetric division, which does not reveal the full picture. A stochastic treatment is needed to be able to incorporate population-level strategies such as a combination of both asymmetric and symmetric division and differentiation. Our work also links with the idea of a potential landscape of cell states [67] (although here, the axes of the landscape represent not, say, expression levels of a protein, but numbers of cells in each sub-population): one simulation represents a niche lineage moving along the landscape and falling into a stable state (the homeostatic state for that lineage), and many simulations, as we have done, could allow us to reconstruct the potential landscape by randomly generating trajectories until we can see its full shape. Thus Monte Carlo simulations offer a computational way to explore the potential landscape.

In this paper, we first introduced a fast method of simulating an entire metapopulation of interacting niche lineages (or cells or biochemical species) synchronously through time. This is based on a version of the 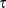-leap method [51] and then generalised to the metapopulation level. It compares favourably with the popular stochastic simulation algorithm method [48], both in terms of speed and accuracy – when interactions are to be included, the stochastic simulation algorithm averages them over time, as each member of the population proceeds through time at a different pace. The computational method we have proposed here can be combined with many stochastic simulation schemes in order to allow one to quickly and easily simulate whole metapopulations. Naturally, it is not limited to cell metapopulations, and can be used in *any* context where we would otherwise use Gillespie's standard SSA to simulate biochemical populations without tracking individual particles. For instance, with no interactions specified, it can be used to simultaneously run many repeat simulations of the same chemical reaction system (by regarding each sub-simulation as an independent repeat simulation), in order to find the full distribution of possible states, arising from intrinsic noise, at some time. However, it is especially useful when we are interested in interacting populations/metapopulations; for instance, this is often the case in ecological systems. It could also be used in condensed matter and chemical physics and in any biochemical context with spatial homogeneity. Finally, it is a very short logical step away from a spatial stochastic model made up of separate compartments (e.g., [68], [69]), and this is one obvious extension.

We used this method to build upon the haematopoietic stem cell model introduced in [27], to simulate a heterogeneous metapopulation of haematopoietic stem cell lineages in time. Using this model, we considered the coupling of individual niche lineages into niche groups. We found that the more niche lineages are coupled, the more closely the mean blood cell numbers approached the target cell ratio. Moreover, when perturbations affected each lineage randomly, as would be the case in a natural environment, a larger number of niche lineages being coupled leads to a smaller overshoot in cell numbers, implying a more ideal response. This suggests that it is advantageous for an organism to couple haematopoietic lineages in order to better regulate homeostasis in the haematopoietic system, as well as respond better to natural perturbations.

Our work leads naturally on to questions about linking cells into whole tissues [65]; for instance, an obvious question is whether these are evolutionarily favourable compared to single niche lineages (or cells). One advantage might be the ability of larger systems to ‘average out’ excessive noise, as is the case with our coupled niche groups. So far, there are few studies investigating whole populations of stem cells, and even fewer on the consequences of linking them into tissues. It is well-known that HSCs routinely leave the niche and migrate in the bloodstream [19], [57], [70]. Using our current model, an easy modification is to allow for this migration into and out of the niches (which might mitigate the instances of all stem cells in one lineage dying out, as we observed). Another extension of our work would be to introduce environmental or even genetic heterogeneity into the picture. Then it becomes possible to investigate the effects of mutations, for instance by introducing niche lineages with different parameters, in a similar way to evolutionary invasion analysis.

## Supporting Information

Figure S1 **Trajectories of stochastic simulations of all cell species, with six uncoupled and six coupled niche lineages.**
(PDF)

Figure S2
**Trajectories of stochastic simulations of uncoupled and coupled niche lineages.** Shown are six individual lineage A) total 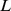 (normalised by niche group size) for six uncoupled and six coupled entire niche groups (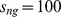) over time; B) trajectories of 100 simulations of uncoupled (top half) and coupled (bottom half), where colour represents the populations of 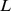 in each niche lineage, and similarly for C), where colour now represents total niche group 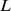, normalised by niche group size.(PDF)

Figure S3
**PDFs of both uncoupled and coupled individual niche lineage** 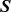 **, for five different MPCR parameter sets.** The axes for each histogram are identical, and quantified on the left and top. MPCR parameters are varied on the bottom axis.(PDF)

Figure S4
**PDFs of both uncoupled and coupled total niche group** 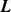 **, for five different MPCR parameter sets.** The axes for each histogram are identical, and quantified on the left and top. MPCR parameters are varied on the bottom axis.(PDF)

Figure S5 **Means and variances of total niche group cell distributions for various MPCR parameter sets.** Distribution means of A) cell types with low numbers; B) cell types with high numbers. Variances of C) cell types with low numbers; D) cell types with high numbers. ODE solutions have been added to A) and B) to show how closely they follow the means of the stochastic distributions.
(PDF)

Figure S6 **Means and variances of feedback distributions for various MPCR parameter sets.** A) Feedback distribution means, B) individual niche lineage variances, and C) total niche group variances for different MPCR parameter sets.
(PDF)

Figure S7
**Steady-state distributions of** 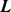 **cell numbers for various niche group sizes.** PDFs of A) individual niche lineage 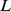 and B) niche group total 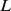, normalised by niche group size, at 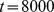 seconds for various niche group sizes. Inset shows the variance of niche group total 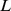 PDFs as a function of niche group size.(PDF)

Figure S8
**Steady-state distributions of feedbacks for various niche group sizes.** PDFs of A) niche group mean MPCR and B) niche group mean 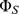 at 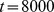 seconds for various niche group sizes.(PDF)

Text S1 **Supporting information text.** Section 1: Deterministic model of the HSC system, with the differential equations listed for each species. Section 2: System parameters and steady states, where the effects of the MPCR and other parameters on the homeostatic cell levels of the system are explored. Section 3: Investigating the target homeostatic cell levels, where we examine whether it is the coupled or uncoupled niche lineages that better find the target cell levels using a different parameter set for the HSC model.
(PDF)

## References

[1] Mason C, Dunnill P (2008) A brief definition of regenerative medicine. Regenerative Medicine 3: 1–5. 18154457 10.2217/17460751.3.1.1

[2] Wagers AJ (2012) The stem cell niche in regenerative medicine. Cell Stem Cell 10: 362–369. 22482502 10.1016/j.stem.2012.02.018

[3] Weissman IL (2000) Translating stem and progenitor cell biology to the clinic: barriers and opportunities. Science 287: 14421446. 10.1126/science.287.5457.1442 10688785

[4] Gurtner GC, Callaghan MJ, Longaker MT (2007) Progress and potential for regenerative medicine. Annual Review of Medicine 58: 299–312. 10.1146/annurev.med.58.082405.095329 17076602

[5] Till JE, McCulloch EA, Siminovitch L (1964) A stochastic model of stem cell proliferation, based on the growth of spleen colony-forming cells. PNAS 51: 29–36. 14104600 10.1073/pnas.51.1.29 PMC300599

[6] Fuchs E, Tumbar T, Guasch G (2004) Socializing with the neighbors: stem cells and their niche. Cell 116: 769778. 10.1016/s0092-8674(04)00255-7 15035980

[7] Metcalf D (2007) Concise review: hematopoietic stem cells and tissue stem cells: current concepts and unanswered questions. Stem Cells 25: 2390–2395. 17690176 10.1634/stemcells.2007-0544

[8] Scadden DT (2006) The stem-cell niche as an entity of action. Nature 441: 1075–1079. 16810242 10.1038/nature04957

[9] Lander AD (2009) The stem cell concept: is it holding us back? Journal of Biology 8: 70. 19769787 10.1186/jbiol177 PMC2776917

[10] Peerani R, Rao BM, Bauwens C, Yin T, Wood GA, et al (2007) Niche-mediated control of human embryonic stem cell self-renewal and differentiation. EMBO Journal 26: 4744–4755. 17948051 10.1038/sj.emboj.7601896 PMC2080799

[11] Stine RR, Matunis EL (2013) Stem cell competition: finding balance in the niche. Trends in Cell Biology 23: 357–364. 23597843 10.1016/j.tcb.2013.03.001 PMC3729747

[12] Hsu YC, Fuchs E (2012) A family business: stem cell progeny join the niche to regulate homeostasis. Nature Reviews Molecular Cell Biology 13: 103–114. 22266760 10.1038/nrm3272 PMC3280338

[13] Takeda N, Jain R, LeBoeuf MR, Wang Q, Lu MM, et al (2011) Interconversion between intestinal stem cell populations in distinct niches. Science 334: 1420–1424. 22075725 10.1126/science.1213214 PMC3705713

[14] Orkin SH, Zon LI (2008) Hematopoiesis: an evolving paradigm for stem cell biology. Cell 132: 631644. 10.1016/j.cell.2008.01.025 PMC2628169 18295580

[15] Mangel M, Bonsall MB (2008) Phenotypic evolutionary models in stem cell biology: replacement, quiescence, and variability. PLoS ONE 3: e1591. 18270578 10.1371/journal.pone.0001591 PMC2217616

[16] Lo Celso C, Scadden DT (2011) The haematopoietic stem cell niche at a glance. Journal of Cell Science 124: 35293535. 10.1242/jcs.074112 PMC3215569 22083139

[17] Morrison SJ, Spradling AC (2008) Stem cells and niches: mechanisms that promote stem cell maintenance throughout life. Cell 132: 598611. 10.1016/j.cell.2008.01.038 PMC4505728 18295578

[18] Kiel MJ, Morrison SJ (2008) Uncertainty in the niches that maintain haematopoietic stem cells. Nature Reviews Immunology 8: 290–301. 10.1038/nri2279 18323850

[19] Yin T, Li L (2006) The stem cell niches in bone. Journal of Clinical Investigation 116: 11951201. 10.1172/JCI28568 PMC1451221 16670760

[20] Morrison SJ, Scadden DT (2014) The bone marrow niche for haematopoietic stem cells. Nature 505: 327–334. 24429631 10.1038/nature12984 PMC4514480

[21] Kiel MJ, Yilmaz OH, Iwashita T, Terhorst C, Morrison SJ (2005) Slam family receptors distinguish hematopoietic stem and progenitor cells and reveal endothelial niches for stem cells. Cell 121: 11091121. 10.1016/j.cell.2005.05.026 15989959

[22] Sugiyama T, Kohara H, Noda M, Nagasawa T (2006) Maintenance of the hematopoietic stem cell pool by cxcl12-cxcr4 chemokine signaling in bone marrow stromal cell niches. Immunity 25: 977988. 10.1016/j.immuni.2006.10.016 17174120

[23] Wang L, Benedito R, Bixel MG, Zeuschner D, Stehling M, et al (2013) Identification of a clonally expanding haematopoietic compartment in bone marrow. EMBO Journal 32: 219–230. 23188081 10.1038/emboj.2012.308 PMC3553379

[24] Hawkins ED, Lo Celso C (2013) Subdivision of bone marrow microenvironments: purpose built homes for haematopoietic stem cells. EMBO Journal 32: 176–177. 23249987 10.1038/emboj.2012.337 PMC3553387

[25] Metcalf D (1988) The Molecular Control of Blood Cells. Cambridge, MA: Harvard University Press.

[26] Mangel M, Bonsall MB (2007) The evolutionary ecology of stem cells and their niches - the time is now. Oikos 116: 17791781.

[27] Mangel M, Bonsall MB (2013) Stem cell biology is population biology: differentiation of hematopoietic multipotent progenitors to common lymphoid and myeloid progenitors. Theoretical Biology and Medical Modelling 10: 5. 23327512 10.1186/1742-4682-10-5 PMC3765094

[28] Lander AD, Gokoffski KK, Wan FYM, Nie Q, Calof AL (2009) Cell lineages and the logic of proliferative control. PLoS Biology 7: e1000015. 10.1371/journal.pbio.1000015 PMC2628408 19166268

[29] Cheshier SH, Prohaska SS, Weissman IL (2007) The effect of bleeding on hematopoietic stem cell cycling and self-renewal. Stem Cells and Development 16: 707–717. 17999593 10.1089/scd.2007.0017

[30] de Graaf CA, Kauppi M, Baldwin T, Hyland CD, Metcalf D, et al (2010) Regulation of hematopoietic stem cells by their mature progeny. PNAS 107: 2168921694. 10.1073/pnas.1016166108 PMC3003054 21115812

[31] Ogawa M (1993) Differentiation and proliferation of hematopoietic stem cells. Blood 81: 2844–2853. 8499622

[32] Huang S (2011) Systems biology of stem cells: three useful perspectives to help overcome the paradigm of linear pathways. Philosophical Transactions of the Royal Society B: Biological Sciences 366: 2247–2259. 10.1098/rstb.2011.0008 PMC3130416 21727130

[33] Osafune K, Caron L, Borowiak M, Martinez RJ, Fitz-Gerald CS, et al (2008) Marked differences in differentiation propensity among human embryonic stem cell lines. Nature Biotechnology 26: 313–315. 10.1038/nbt1383 18278034

[34] Sieburg HB, Rezner BD, Muller-Sieburg CE (2011) Predicting clonal self-renewal and extinction of hematopoietic stem cells. PNAS 108: 43704375. 10.1073/pnas.1011414108 PMC3060234 21368169

[35] Huang S (2009) Non-genetic heterogeneity of cells in development: more than just noise. Development 136: 38533862. 10.1242/dev.035139 PMC2778736 19906852

[36] Lander AD (2011) The individuality of stem cells. BMC Biology 9: 40. 21649941 10.1186/1741-7007-9-40 PMC3110137

[37] Suda T, Suda J, Ogawa M (1983) Single-cell origin of mouse hemopoietic colonies expressing multiple lineages in variable combinations. PNAS 80: 66896693. 10.1073/pnas.80.21.6689 PMC391236 6579554

[38] Marciniak-Czochra A, Stiehl T, Ho AD, Jäger W, Wagner W (2009) Modeling of asymmetric cell division in hematopoietic stem cells – regulation of self-renewal is essential for efficient repopulation. Stem Cells and Development 18: 377–385. 18752377 10.1089/scd.2008.0143

[39] Dingli D, Pacheco JM (2011) Stochastic dynamics and the evolution of mutations in stem cells. BMC Biology 9: 41. 21649942 10.1186/1741-7007-9-41 PMC3110138

[40] Gupta PB, Fillmore CM, Jiang G, Shapira SD, Tao K, et al (2011) Stochastic state transitions give rise to phenotypic equilibrium in populations of cancer cells. Cell 146: 633–644. 21854987 10.1016/j.cell.2011.07.026

[41] Sun Z, Komarova NL (2012) Stochastic modeling of stem-cell dynamics with control. Mathematical Biosciences 240: 231240. 10.1016/j.mbs.2012.08.004 PMC3921979 22960597

[42] Komarova NL (2013) Principles of regulation of self-renewing cell lineages. PLoS ONE 8: e72847. 24019882 10.1371/journal.pone.0072847 PMC3760876

[43] Weissman IL (2000) Stem cells: units of development, units of regeneration, and units in evolution. Cell 100: 157–168. 10647940 10.1016/s0092-8674(00)81692-x

[44] Laird DJ, Tomaso AWD, Weissman IL (2005) Stem cells are units of natural selection in a colonial ascidian. Cell 123: 1351–1360. 16377573 10.1016/j.cell.2005.10.026

[45] Gillespie DT, Mangel M (1981) Conditioned averages in chemical kinetics. Journal of Chemical Physics 75: 704–709.

[46] Goutsias J (2007) Classical versus stochastic kinetics modeling of biochemical reaction systems. Biophysical Journal 92: 2350–2365. 17218456 10.1529/biophysj.106.093781 PMC1864832

[47] Wilkinson DJ (2009) Stochastic modelling for quantitative description of heterogeneous biological systems. Nature Reviews Genetics 10: 122–133. 10.1038/nrg2509 19139763

[48] Gillespie DT (1977) Exact stochastic simulation of coupled chemical reactions. Journal of Physical Chemistry 81: 2340–2361.

[49] Mangel M (2006) The Theoretical Biologist's Toolbox: Quantitative Methods for Ecology and Evolutionary Biology. Cambridge, UK: Cambridge University Press.

[50] Gillespie DT (2009) A diffusional bimolecular propensity function. Journal of Chemical Physics 131: 164109. 19894929 10.1063/1.3253798 PMC2780463

[51] Gillespie DT (2001) Approximate accelerated stochastic simulation of chemically reacting systems. Journal of Chemical Physics 115: 1716–1733.

[52] Cao Y, Gillespie DT, Petzold LR (2006) Efficient step size selection for the tau-leaping simulation method. Journal of Chemical Physics 124: 044109. 16460151 10.1063/1.2159468

[53] Hu Y, Li T, Min B (2011) A weak second order tau-leaping method for chemical kinetic systems. Journal of Chemical Physics 135: 024113. 21766931 10.1063/1.3609119

[54] Székely T Jr, Burrage K, Erban R, Zygalakis KC (2012) A higher-order numerical framework for stochastic simulation of chemical reaction systems. BMC Systems Biology 6: 85. 23256696 10.1186/1752-0509-6-85 PMC3529698

[55] Székely T Jr, Burrage K, Zygalakis KC, Barrio M (2014) Efficient simulation of stochastic chemical kinetics with the Stochastic Bulirsch-Stoer extrapolation method. BMC Systems Biology 8: 71. 24939084 10.1186/1752-0509-8-71 PMC4085235

[56] Kurtz TG (1978) Strong approximation theorems for density dependent markov chains. Stochastic Processes and their Applications 6: 223–240.

[57] Wright DE, Wagers AJ, Gulati AP, Johnson FL, Weissman IL (2001) Physiological migration of hematopoietic stem and progenitor cells. Science 294: 1933–1936. 11729320 10.1126/science.1064081

[58] Adams GB, Scadden DT (2006) The hematopoietic stem cell in its place. Nature Immunology 7: 333–337. 16550195 10.1038/ni1331

[59] Colijn C, Mackey MC (2005) A mathematical model of hematopoiesis - I. periodic chronic myelogenous leukemia. Journal of Theoretical Biology 237: 117–132. 15975596 10.1016/j.jtbi.2005.03.033

[60] Fallen PR, McGreavey L, Madrigal JA, Potter M, Ethell M, et al (2003) Factors affecting reconstitution of the T cell compartment in allogeneic haematopoietic cell transplant recipients. Bone Marrow Transplantation 32: 1001–1014. 14595388 10.1038/sj.bmt.1704235

[61] Dumont-Girard F, Roux E, van Lier RA, Hale G, Helg C, et al (1998) Reconstitution of the T-cell compartment after bone marrow transplantation: restoration of the repertoire by thymic emigrants. Blood 92: 4464–4471. 9834254

[62] Sieburg HB, Cho RH, Dykstra B, Uchida N, Eaves CJ, et al (2006) The hematopoietic stem compartment consists of a limited number of discrete stem cell subsets. Blood 107: 2311–2316. 16291588 10.1182/blood-2005-07-2970 PMC1456063

[63] Cheshier SH, Morrison SJ, Liao X, Weissman IL (1999) In vivo proliferation and cell cycle kinetics of long-term self-renewing hematopoietic stem cells. PNAS 96: 3120–3125. 10077647 10.1073/pnas.96.6.3120 PMC15905

[64] Morrison SJ, Kimble J (2006) Asymmetric and symmetric stem-cell divisions in development and cancer. Nature 441: 1068–1074. 16810241 10.1038/nature04956

[65] O'Brien LE, Bilder D (2013) Beyond the niche: tissue-level coordination of stem cell dynamics. Annual Review of Cell and Developmental Biology 29: 107–136. 10.1146/annurev-cellbio-101512-122319 PMC3897713 23937350

[66] Rodriguez-Brenes IA, Komarova NL, Wodarz D (2011) Evolutionary dynamics of feedback escape and the development of stem-cell-driven cancers. PNAS 108: 18983–18988. 22084071 10.1073/pnas.1107621108 PMC3223454

[67] Wang J, Wang E, Huang S (2010) The potential landscape of genetic circuits imposes the arrow of time in stem cell differentiation. Biophysical Journal 99: 29–39. 20655830 10.1016/j.bpj.2010.03.058 PMC2895388

[68] Hattne J, Fange D, Elf J (2005) Stochastic reaction-diffusion simulation with MesoRD. Bioinformatics 21: 2923–2924. 15817692 10.1093/bioinformatics/bti431

[69] Marquez-Lago T, Burrage K (2007) Binomial tau-leap spatial stochastic simulation algorithm for applications in chemical kinetics. Journal of Chemical Physics 127: 104101. 17867731 10.1063/1.2771548

[70] Bhattacharya D, Czechowicz A, Ooi AG, Rossi DJ, Bryder D, et al (2009) Niche recycling through division-independent egress of hematopoietic stem cells. Journal of Experimental Medicine 206: 2837–2850. 19887396 10.1084/jem.20090778 PMC2806613

