# Supplementary figures and images for: Stochastic Dynamics of Interacting Haematopoietic Stem Cell Niche Lineages
Source: PLoS Comput Biol. 2014 Sep 4;10(9):e1003794. doi: 10.1371/journal.pcbi.1003794 (PMC4154659; doi:10.1371/journal.pcbi.1003794)

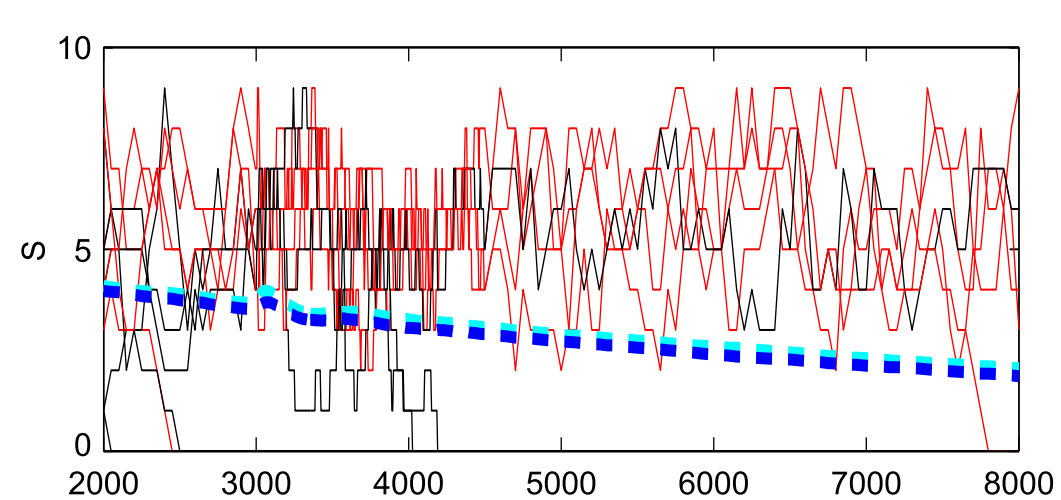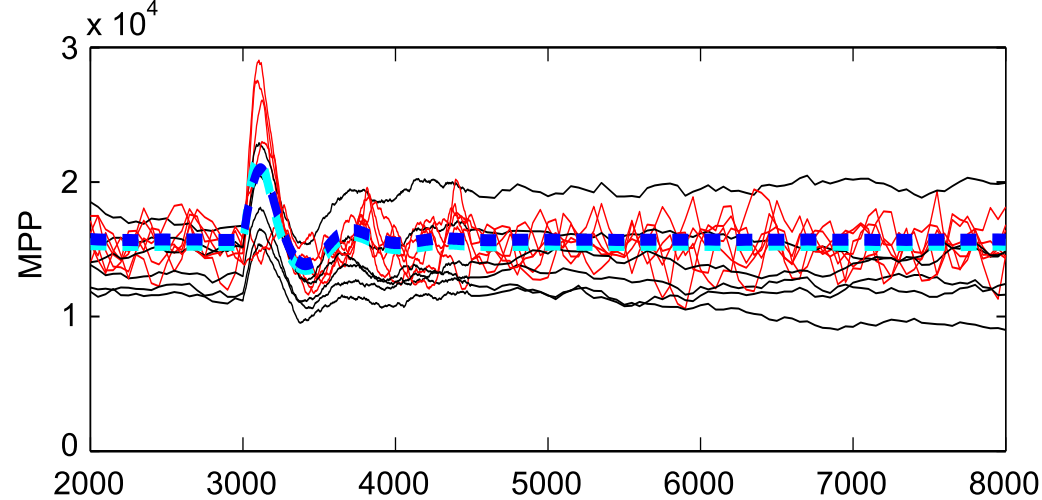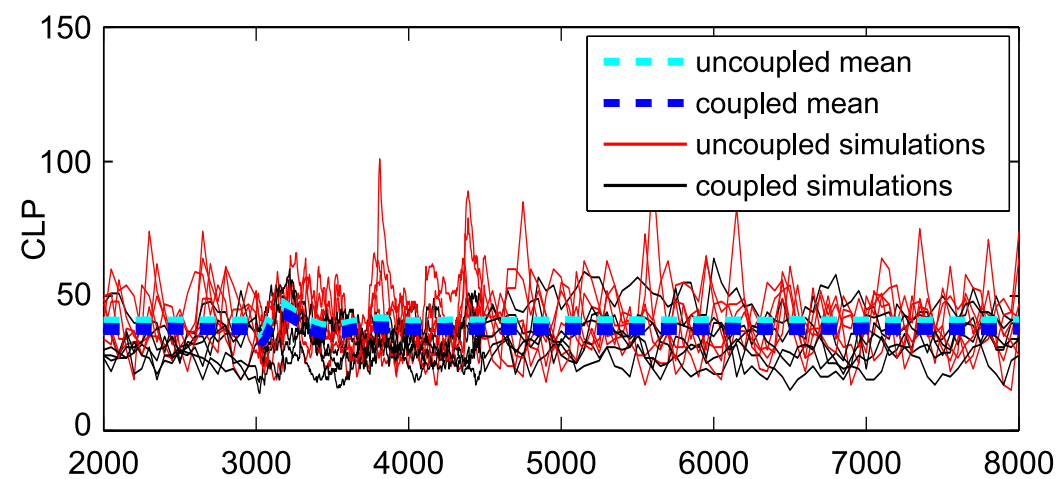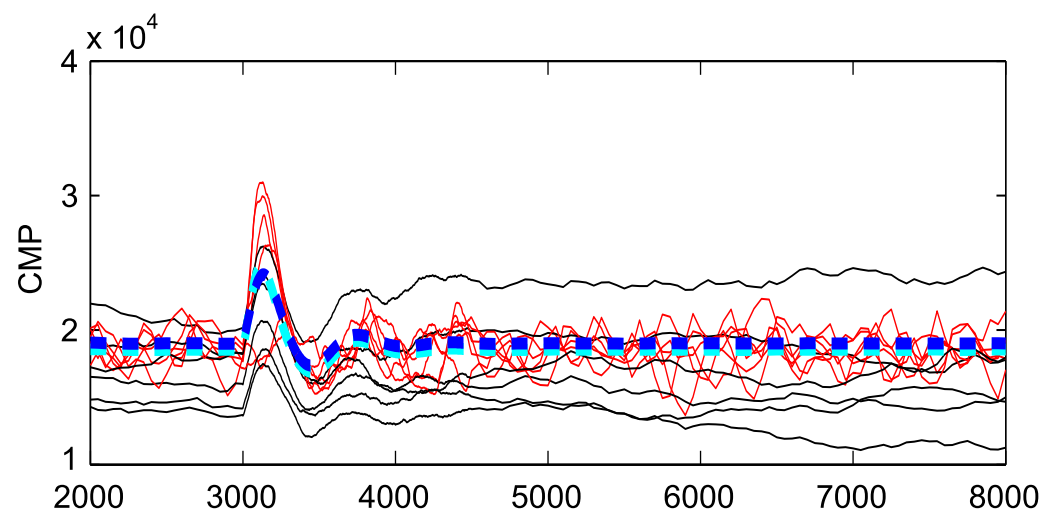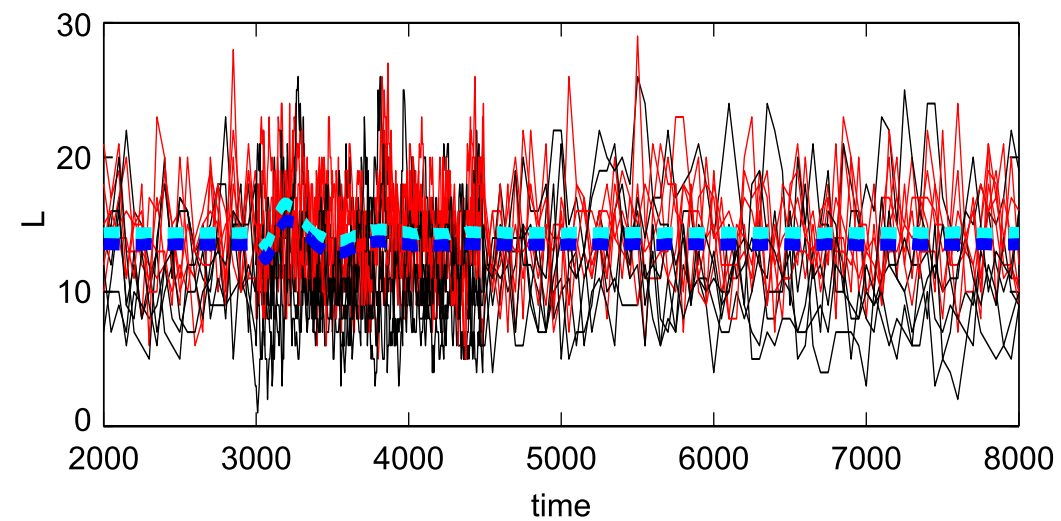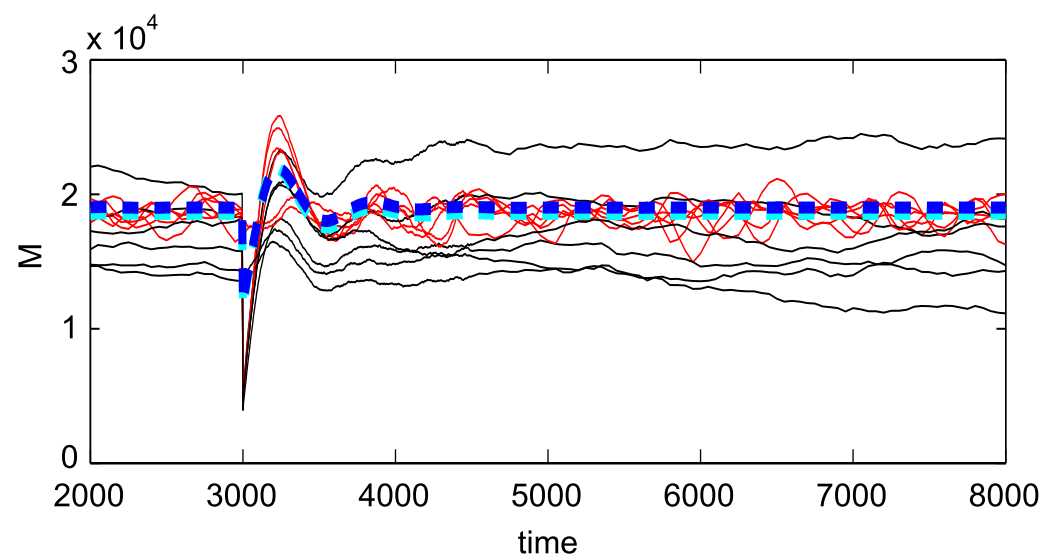

Supplement: Figure S1 — Trajectories of stochastic simulations of all cell species, with six uncoupled and six coupled niche lineages. (PDF) [file pcbi.1003794.s001.pdf]

**A**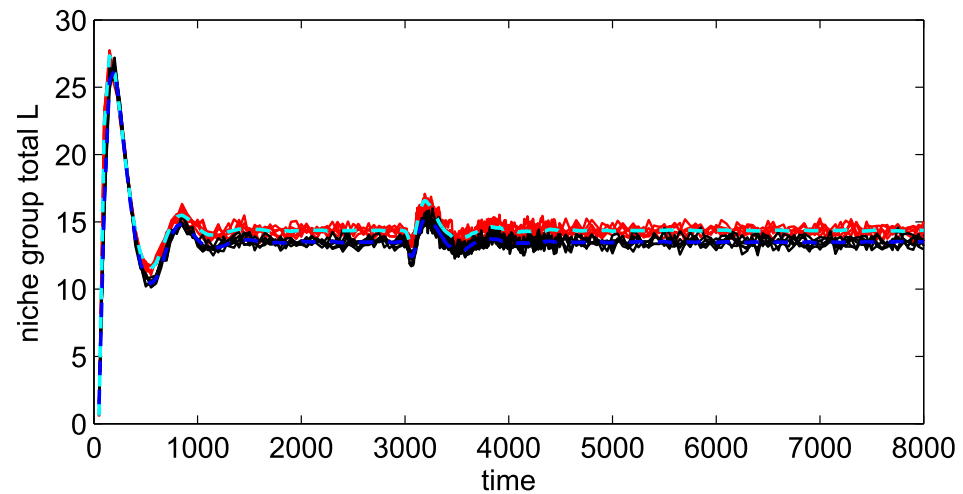**B**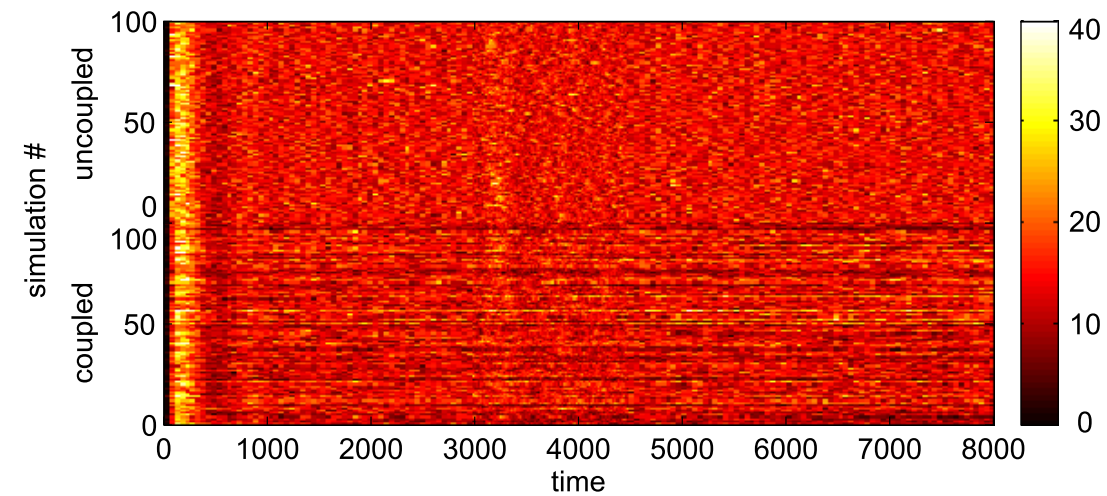**C**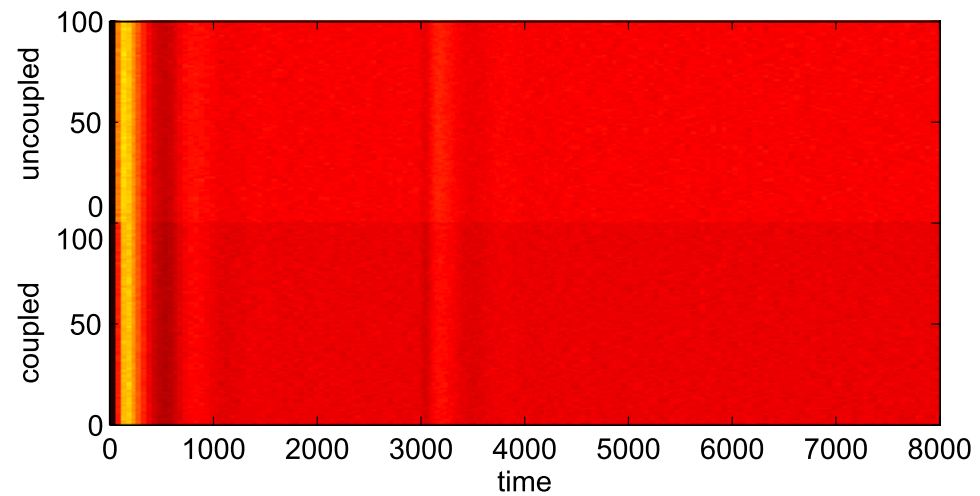

Supplement: Figure S2 — Trajectories of stochastic simulations of uncoupled and coupled niche lineages. Shown are six individual lineage A) total (normalised by niche group size) for six uncoupled and six coupled entire niche groups () over time; B) trajectories of 100 simulations of uncoupled (top half) and coupled (bottom half), where colour represents the populations of in each niche lineage, and similarly for C), where colour now represents total niche group , normalised by niche group size. (PDF) [file pcbi.1003794.s002.pdf]

$p(S)$

0.5 0.25 0 0.25 0.5

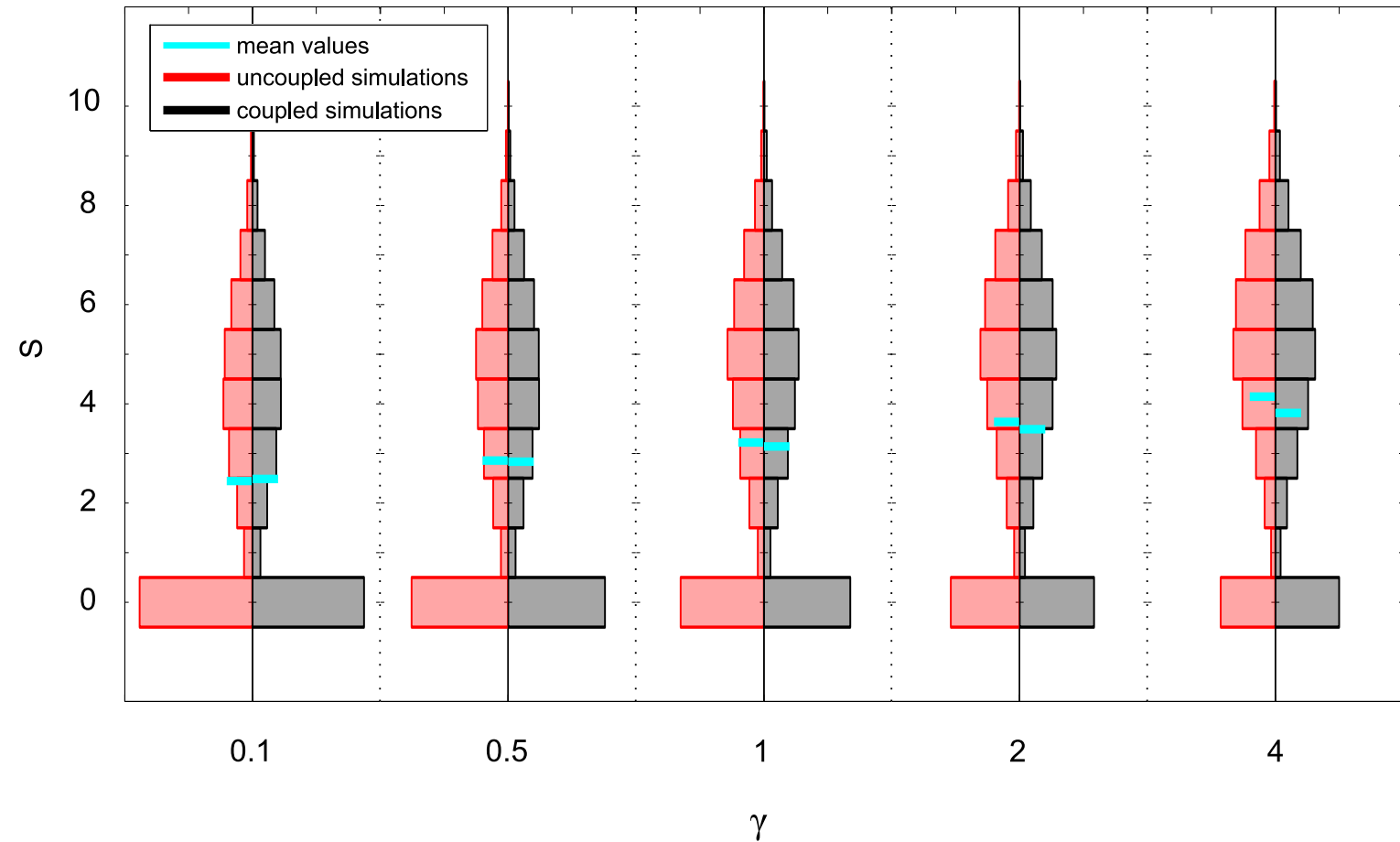

Supplement: Figure S3 — PDFs of both uncoupled and coupled individual niche lineage , for five different MPCR parameter sets. The axes for each histogram are identical, and quantified on the left and top. MPCR parameters are varied on the bottom axis. (PDF) [file pcbi.1003794.s003.pdf]

p(total L)

1.2 0.6 0 0.6 1.2

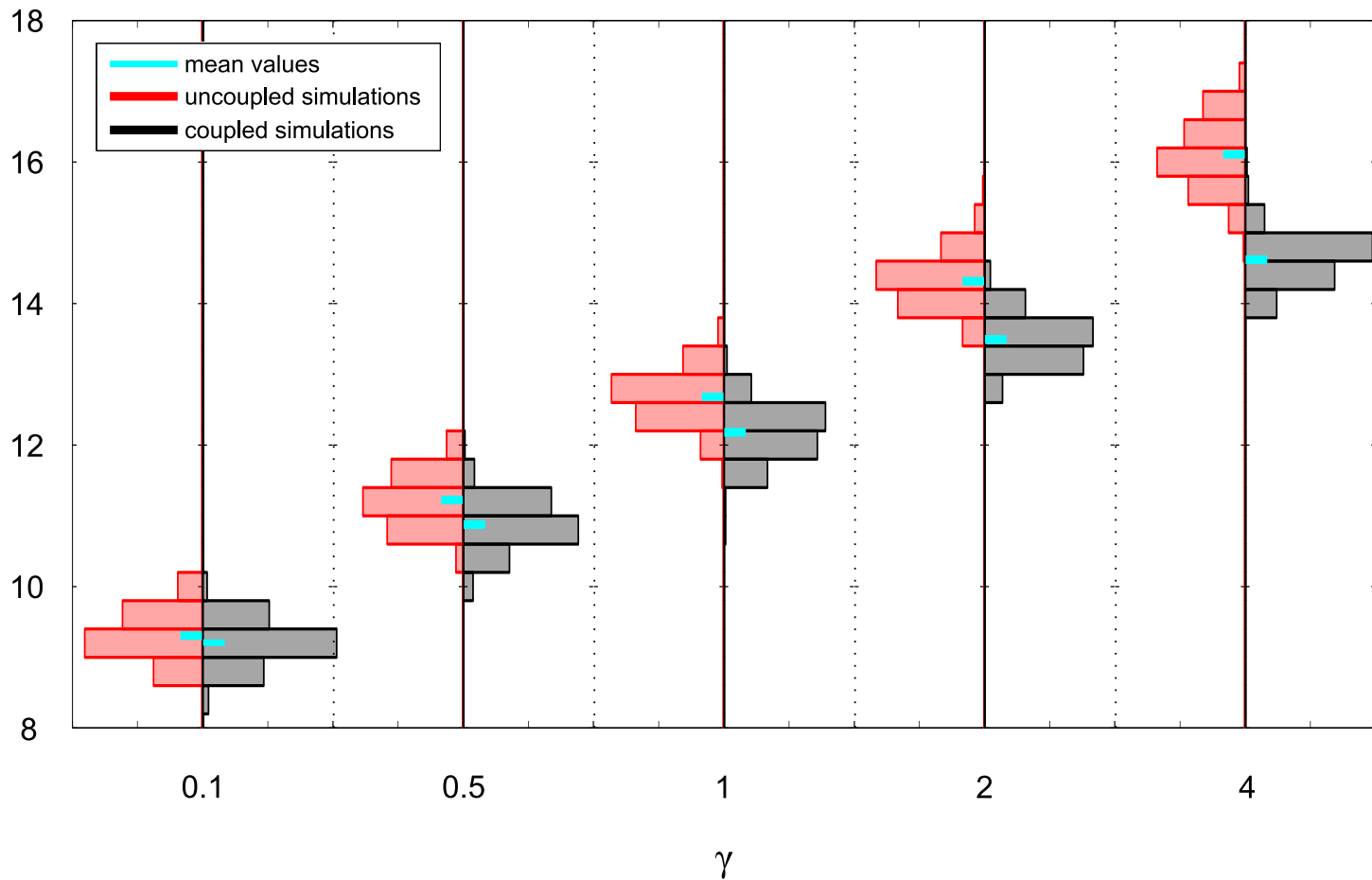

Supplement: Figure S4 — PDFs of both uncoupled and coupled total niche group , for five different MPCR parameter sets. The axes for each histogram are identical, and quantified on the left and top. MPCR parameters are varied on the bottom axis. (PDF) [file pcbi.1003794.s004.pdf]

A

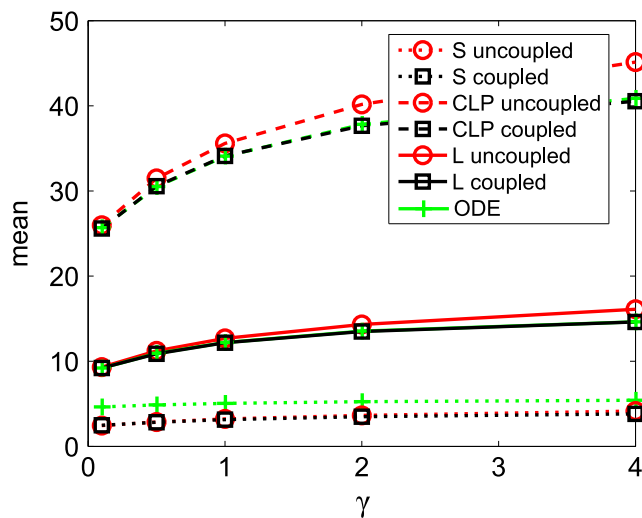

B

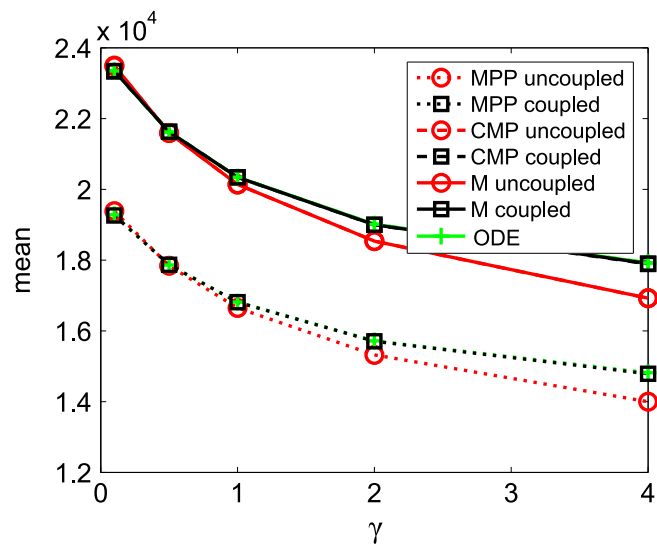

C

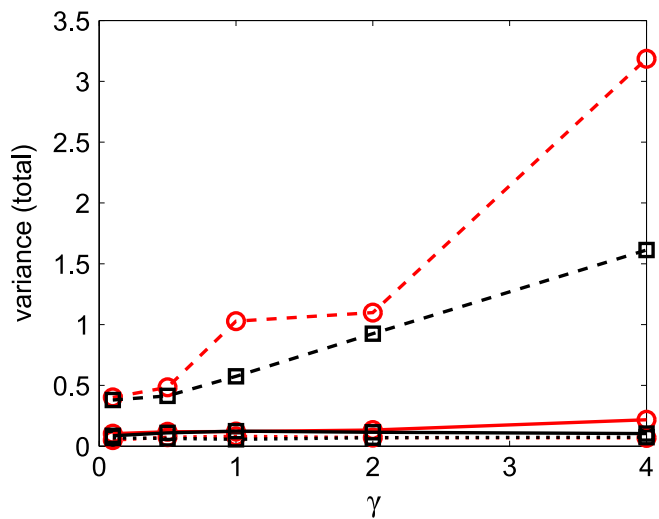

D

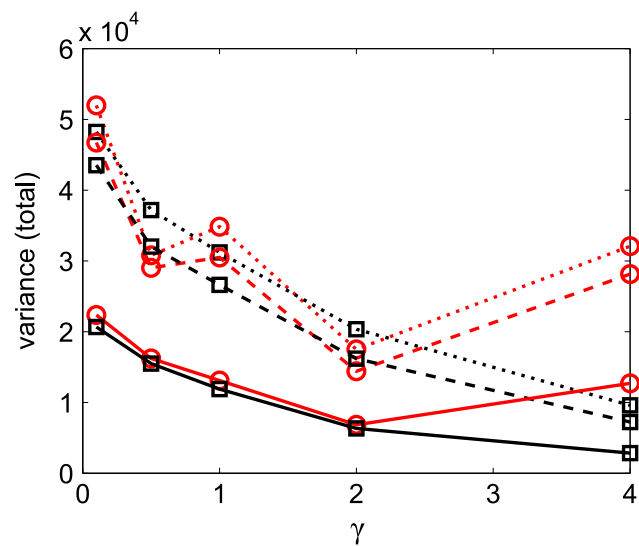

Supplement: Figure S5 — Means and variances of total niche group cell distributions for various MPCR parameter sets. Distribution means of A) cell types with low numbers; B) cell types with high numbers. Variances of C) cell types with low numbers; D) cell types with high numbers. ODE solutions have been added to A) and B) to show how closely they follow the means of the stochastic distributions. (PDF) [file pcbi.1003794.s005.pdf]

**A**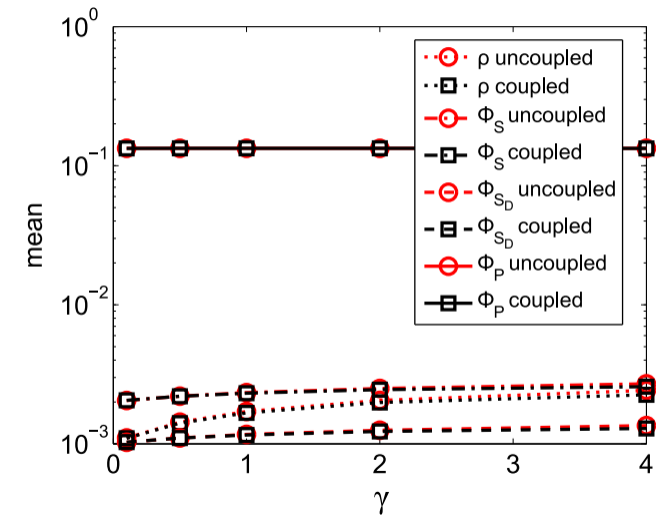**B**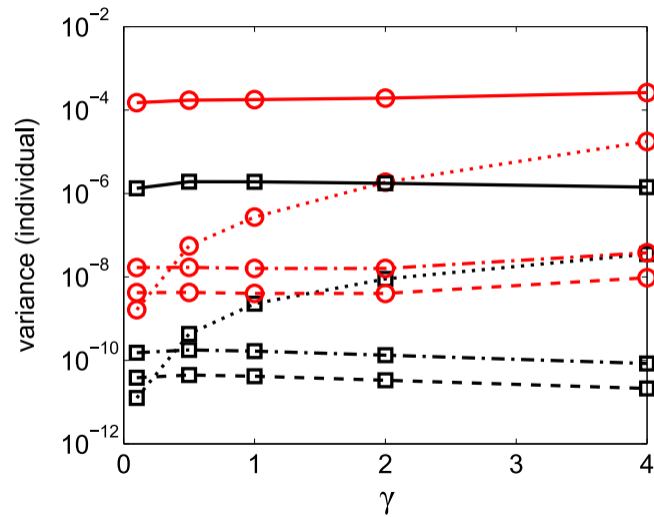**C**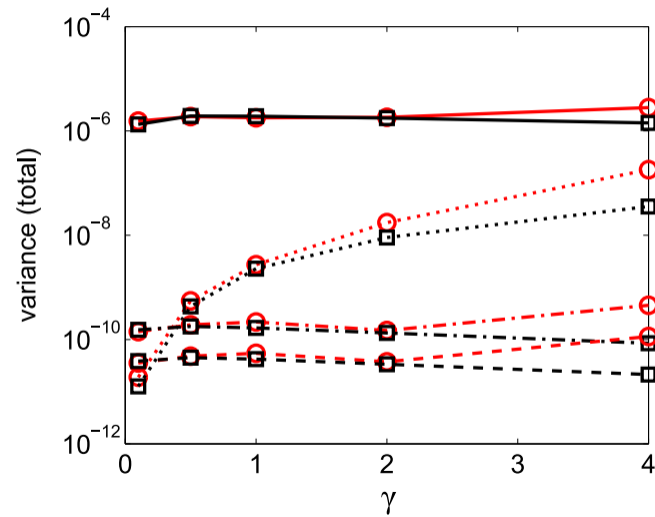

Supplement: Figure S6 — Means and variances of feedback distributions for various MPCR parameter sets. A) Feedback distribution means, B) individual niche lineage variances, and C) total niche group variances for different MPCR parameter sets. (PDF) [file pcbi.1003794.s006.pdf]

A

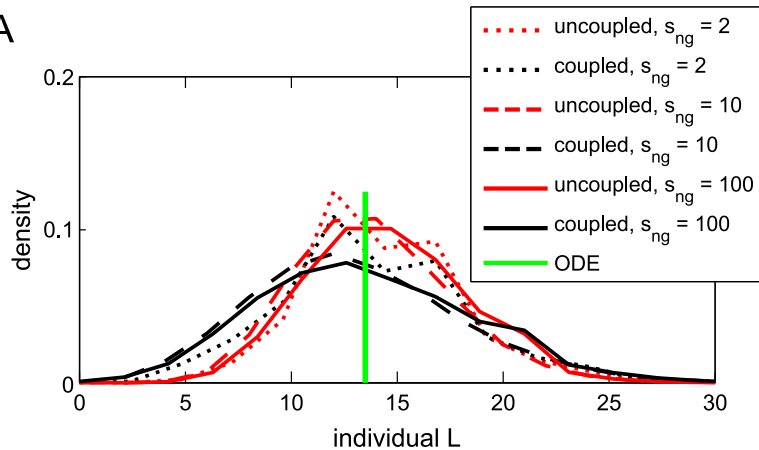

B

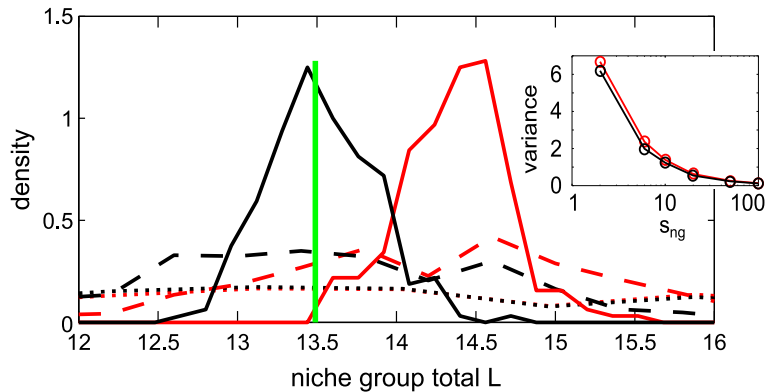

Supplement: Figure S7 — Steady-state distributions of cell numbers for various niche group sizes. PDFs of A) individual niche lineage and B) niche group total , normalised by niche group size, at seconds for various niche group sizes. Inset shows the variance of niche group total PDFs as a function of niche group size. (PDF) [file pcbi.1003794.s007.pdf]

A

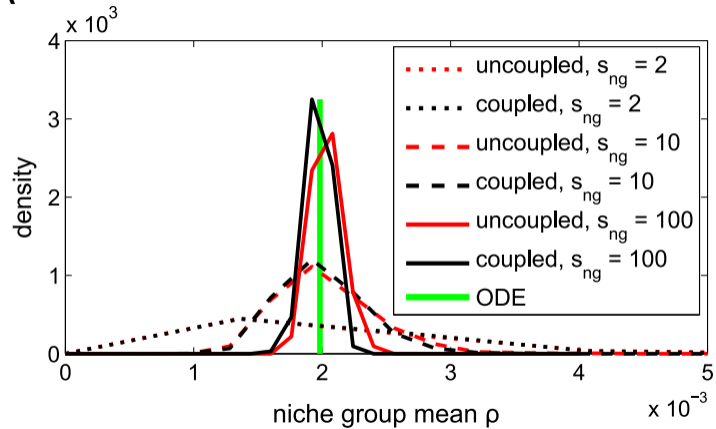

B

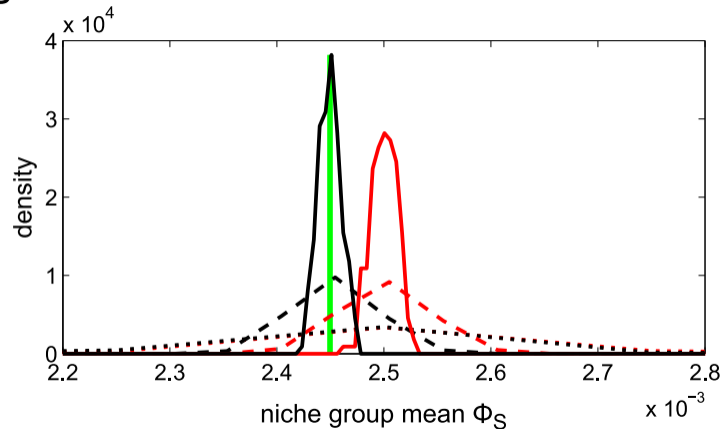

Supplement: Figure S8 — Steady-state distributions of feedbacks for various niche group sizes. PDFs of A) niche group mean MPCR and B) niche group mean at seconds for various niche group sizes. (PDF) [file pcbi.1003794.s008.pdf]
