# Supplementary material for: Stochastic Dynamics of Interacting Haematopoietic Stem Cell Niche Lineages
Source: PLoS Comput Biol. 2014 Sep 4;10(9):e1003794. doi: 10.1371/journal.pcbi.1003794 (PMC4154659; doi:10.1371/journal.pcbi.1003794)
Supplement: Text S1 — Supporting information text. Section 1: Deterministic model of the HSC system, with the differential equations listed for each species. Section 2: System parameters and steady states, where the effects of the MPCR and other parameters on the homeostatic cell levels of the system are explored. Section 3: Investigating the target homeostatic cell levels, where we examine whether it is the coupled or uncoupled niche lineages that better find the target cell levels using a different parameter set for the HSC model. (PDF) [file pcbi.1003794.s009.pdf]

Stochastic Dynamics of Interacting Haematopoietic Stem Cell Niche Lineages

Supporting Text S1

Tamás Székely Jr.[1,*], Kevin Burrage[1,2], Marc Mangel[3,4], Michael B. Bonsall[5]

1 Department of Computer Science, University of Oxford, Oxford, United Kingdom
2 Department of Mathematics, Queensland University of Technology, Brisbane, Queensland, Australia
3 Department of Applied Mathematics and Statistics, University of California Santa Cruz, Santa Cruz, California, United States of America
4 Department of Biology, University of Bergen, Bergen, Norway
5 Mathematical Ecology Research Group, Department of Zoology, University of Oxford, Oxford, United Kingdom

∗ E-mail: tamas.szekely@cs.ox.ac.uk

1 Deterministic model of HSC system

In some of our results below and in the main text, we have used the solutions of the deterministic (ordinary differential equation, ODE) representation of the HSC system. This formulation is thoroughly described in [1], although for this paper we have made some minor modifications. Using the notation from the rest of this paper, the formulae are as follows:

$$\begin{aligned}
\frac{d[S]}{dt} &= \big(r_{S_S}\ln(\tfrac{K}{[S]}) - r_{S_D}\Phi_{S_D}([L],[M])\big)\Phi_S([L],[M])\cdot[S] - \mu_S[S]\\
\frac{d[MPP]}{dt} &= \big(r_{S_A} + 2r_{S_D}\Phi_{S_D}([L],[M])\big)\Phi_S([L],[M])\cdot[S] + (\lambda_P - r_P)\Phi_P([L],[M])\cdot[MPP] - \mu_p[MPP]\\
\frac{d[CLP]}{dt} &= r_P\rho([L],[M])\Phi_P([L],[M])\cdot[MPP] - r_{CLP}[CLP] - \mu_{CLP}[CLP]\\
\frac{d[CMP]}{dt} &= r_P\big(1-\rho([L],[M])\big)\Phi_P([L],[M])\cdot[MPP] - r_{CMP}[CMP] - \mu_{CMP}[CMP]\\
\frac{d[L]}{dt} &= r_{CLP}[CLP] - \mu_L[L]\\
\frac{d[M]}{dt} &= r_{CMP}[CMP] - \mu_M[M]
\end{aligned}$$

The chemical species are in braces to denote concentrations, rather than actual population numbers as in the stochastic model. The rest of the notation and parameters are described fully in Table 2 of the main text.

These equations can be easily solved using a standard ODE solver to find the cell species concentrations over time, as well as at steady state.

2 System parameters and steady states

The MPCR parameters (γ, α) as well as the transition rate constants (r_{S_S}, r_P, μ_L, etc.) also affect the steady states of both the cell levels and feedbacks. As is summarised in Fig.S5, their distributions are shifted, either up or down depending on cell or feedback type, as certain parameters (in this case, γ and α) are varied. Here, we focus on the interactions of the MPCR parameters and the constants $r_{CLP}, r_{CMP}, \mu_{CLP}, \mu_{CMP}, \mu_L, \mu_M$, i.e. the differentiation and death rates of committed progenitors and blood cells, and their role in the cell number steady states.

This phenomenon also occurs using the ODE model (Fig. 1), and the mean values of the stochastic distributions are very close to the ODE solutions (Fig.S5; also Figs. 4 and 6). The only cell type whose

mean levels are not accurately matched by the ODE is S, and this is because the ODEs do not find the absorbing steady state $S = 0$, thus overestimating mean S. Although in general this should not be assumed without careful testing, here we have sufficient evidence that the ODE solutions represent meaningful system properties of the stochastic system (that is, the mean values). Thus in this section, for simplicity we have used the ODE model.

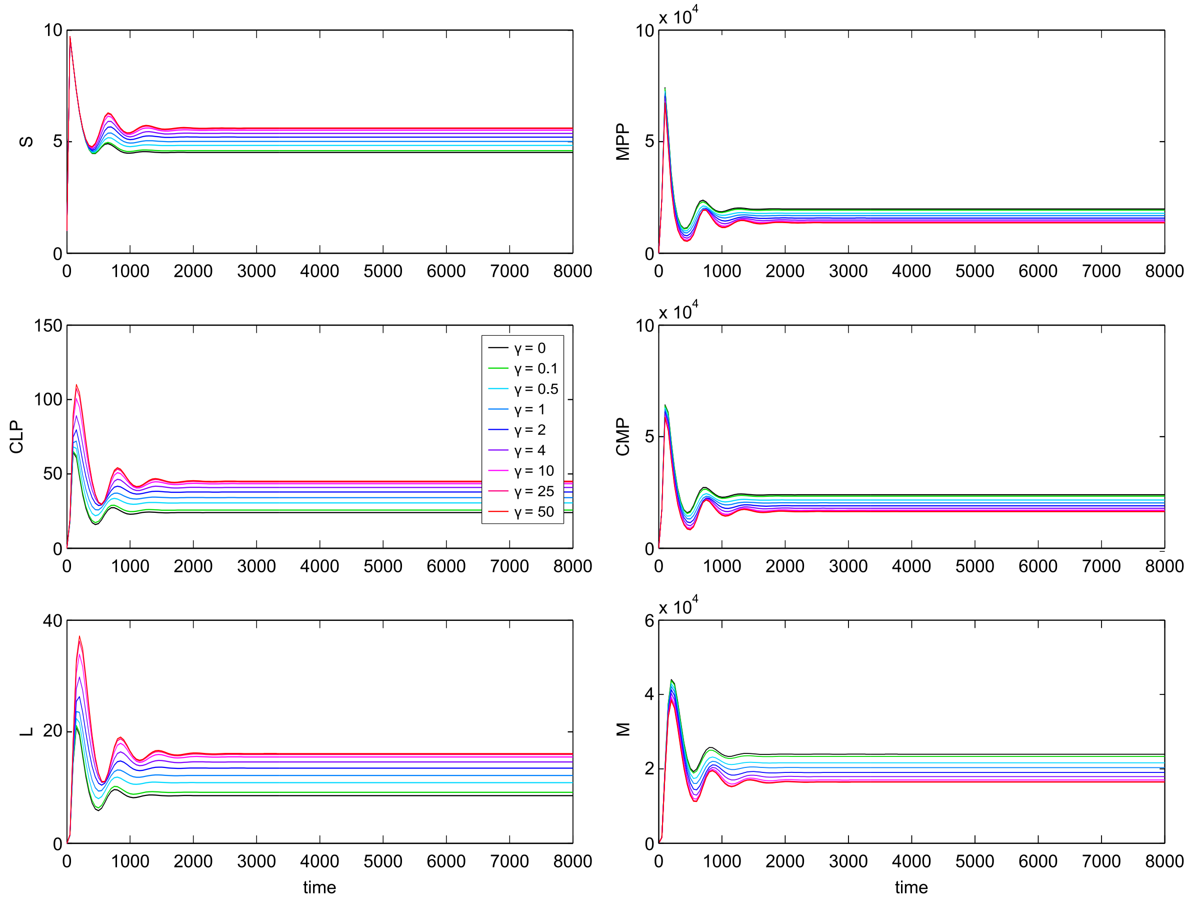

Figure 1. Trajectories of ODE model of HSC system for various MPCR parameters. The desired homeostatic blood cell ratio was set as $1L:1000M$.

We set the homeostatic ratio that we want the cell levels to reach as $\rho_h = 1L:1000M = 9.99 \times 10^{-4}$; thus we also vary α to keep this ratio as γ is changed. We note again the issue with using L, M numbers to set steady state ρ: we must remember that ρ is defined as the probability of an MPP to CLP transition, i.e. $\rho_h = \frac{CLP}{CLP+CMP}$, so it is really the progenitor ratio we are specifying. Writing it instead in terms of L and M, the expression $\rho_h = \frac{L}{L+M}$ is only exact when the lymphoid and myeloid routes are identical

in terms of their differentiation and death rates (otherwise, the system will not reach ρ_h exactly). If we want to use disparate rates, it is possible to take into account the disparities in them when substituting in L, M for CLP, CMP.

However, the situation is only simple when $\gamma = 0$ and the MPCR acts as a constant. What happens as we increase γ, that is make the MPCR more responsive? Starting with $\gamma = 0$, setting $\rho_h = 1 : 1000$ and using the constants given in Table 2 of the main text (notably, $\mu_L = 2.8\mu_M$), the steady states are at $L \approx 8.55, M \approx 2.39 \times 10^4$ (Fig. 1 black line); this is clearly nowhere near $1L\!:\!1000M$. As $8.55 \times 2.8 = 23.9$, it is easy to see how this steady state arises. However, as γ is increased, so the L (and CLP) steady states increase, whilst the M (and CMP) steady states decrease, moving the cell ratios closer and closer to $1L : 1000M$ (because we found γ, α by using this homeostatic L, M ratio in Eq.(2) in the main text). Fig. 1 illustrates γ, α values used in the main text plus some extremes on either side. By the time we reach $\gamma = 50$, a hyper-sensitive response, the steady states have become $L \approx 16.09, M \approx 1.64 \times 10^4$, very close to $1L : 1000M$ – even though the L and M death rates still differ by a factor of 2.8. Thus once $\gamma > 0$, the MPCR responds to the cell levels dynamically by affecting the steady states in a non-trivial way – but always driving them closer to ρ_h as given by Eq.(2) in the main text. As γ is increased, this response becomes stronger and the cell levels change correspondingly. Therefore, this tells us that it is not unreasonable to specify ρ_h using the blood cell, rather than progenitor cell, levels, as the only consequence is a (possible, in the case of asymmetrical parameters) change in the steady state blood cell levels, which is also partly compensated for by the dynamic nature of the MPCR function. This is important, as we want to be able to specify the homeostatic ratio in terms of differentiated blood cell numbers, with the freedom to use asymmetrical parameters in the myeloid and lymphoid routes.

3 Investigating the target homeostatic cell levels

In the Results section of the main text, we discuss the difference in mean cell levels between the uncoupled and coupled niche lineages for the case $\gamma = 2, \alpha = 10^{-9}$. In a somewhat similar way to the effects of the MPCR parameters, the distributions of cell levels and feedbacks are shifted as the niche grouping size is varied. There are also associated changes in their variance, but here we are interested in elucidating which distribution is centred around a more optimal homeostatic state; we explore this in detail below.

First, unlike in the above section, we cannot rely on the deterministic model for help, as without

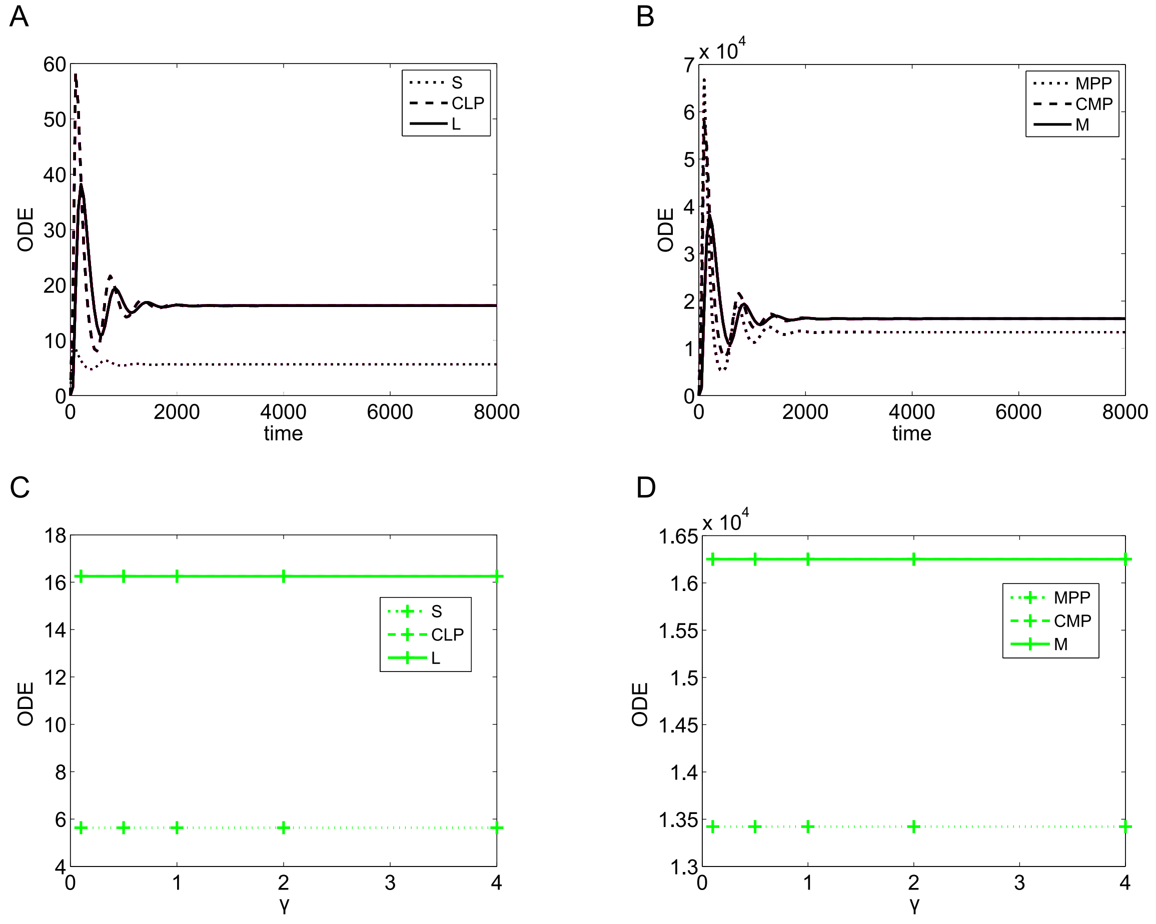

Figure 2. ODE model of HSC system with death rates for L and M cells set identical. This allows the system to reach the same homeostatic cell levels for all MPCR parameters. A) Trajectories of cell types with low numbers, and B) cell types with high numbers for various MPCR parameters (curves for several different γ, α are shown, but they completely overlap because the solutions are identical). C) ODE solutions of cell types with low numbers, and D) cell types with high numbers for various MPCR parameters.

using stochastic simulations we cannot tell whether the ODE solutions represent any useful properties of the true distributions (although Figs. 4 and 6 show that we would be justified in doing this, as the ODEs approximate the mean of the stochastic distributions well).

Unfortunately, stochastic simulations with different niche grouping sizes give different results and we cannot tell which is closer to our target values. The solution to this quandary is to use a set of model parameters where $\mu_L = \mu_M = 0.01$, thus removing the pressure on the dynamical system from these parameters. Since the other parameters are all identical between the lymphoid and myeloid routes

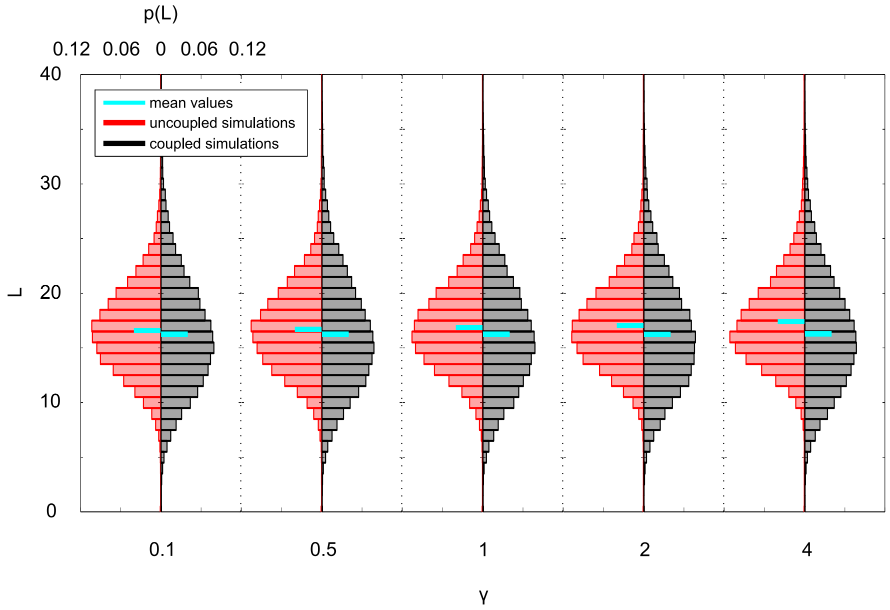

Figure 3. PDFs of both uncoupled and coupled individual niche lineage L, for five different MPCR parameter sets. The death parameters for L and M cells are equal. The axes for each histogram are identical, and quantified on the left and top. MPCR parameters are varied on the bottom axis.

(Table 2, main text), this means that the target steady state levels will exactly follow the ratio $1L\!:\!1000M$ (indeed, the ODE system now finds identical steady states for all γ, α; Fig. 2). We investigate whether it is the coupled or uncoupled lineages that keep their cell levels closer to the target levels ρ_h for several different MPCR parameters.

We illustrate stochastic simulation results for this system in Figs. 3 to 9. Figs. 3 and 4 show the PDFs, both per individual niche lineage and summed over niche groups, of steady state L levels for various γ; similarly, Figs. 5 and 6 show levels of M. Fig. 7 summarises the behaviour of mean L and M. The PDFs of ρ (i.e. the value of the MPCR) are shown in Figs. 8 and 9. From these plots, we can make the following observations:

1. Uncoupled niche lineages have tighter cell level distributions individually, but when summed over niche groups the distributions are similar, with coupled lineages having a slightly smaller variance in almost all cases.

2. For both L and M the distributions get tighter as γ increases (albeit the effect is small for L).

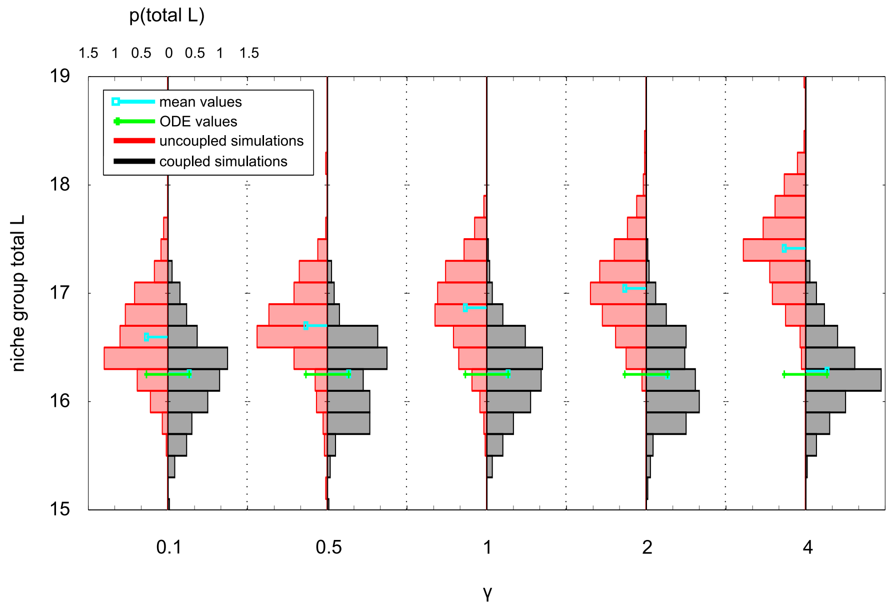

Figure 4. PDFs of both uncoupled and coupled niche group total L, for five different MPCR parameter sets. The ODE values have been marked on to demonstrate their proximity to the mean of the coupled lineages. The niche group totals are normalised by niche group size. The death parameters for L and M cells are equal.

3. The distribution means of the uncoupled lineages vary with γ, but those of the coupled niches change very little. This becomes especially obvious when the blood cell levels are summed over niche groups (Figs. 4 and 6). For easier comparison, Fig. 7 shows mean L and M for several different values of γ.

4. Although the means of the MPCR distributions stay around the expected $\rho_h = 9.99 \times 10^{-4}$ both in the uncoupled and coupled cases (Fig. 8), the coupled lineages achieve a much tighter MPCR distribution than uncoupled ones. This is also true for niche group mean MPCR, although the difference in variance is much smaller (Fig. 9).

5. In addition, as γ increases the bulk of the uncoupled MPCR distribution moves so that it is centred somewhere around 5×10^{-4}, leaving a long tail to still be able to achieve a mean value of ρ_h (Fig. 8).

6. Finally, taking the ratio of the mean cell levels of the uncoupled lineages at $\gamma = 2$ gives an MPCR value of 8.77×10^{-4} compared to 9.99×10^{-4} for the coupled lineages. It is easily visible from Figs. 4 and 6 that at $\gamma = 2$, the distributions of uncoupled L and M have moved away from the target values of $16.25, 1.625 \times 10^4$, respectively.

Putting all this together, especially points 4 to 6, we can conclude that coupling niche lineages allows them to better reach the target homeostatic state, as they are able to keep their blood cell levels very close to the desired $1L : 1000M$ ratio for all γ, as well as more tightly-distributed around those values. Furthermore, coupled niches also keep their MPCR more tightly-distributed around ρ_h than the uncoupled niches. In addition, as we can see from Figs. 4 and 6, the ODE solutions do approximate the stochastic mean values well, in the case of the HSC model.

Thus, we are now in a position to apply the above results to the stochastic system in the main text, where the parameters μ_L and μ_M are not equal. As we have concluded that here the coupled system regulates its cell levels closer to the target level, we can also extrapolate that this is the case for the results in the main text. Furthermore, we have added the ODE solutions to some figures in the main text to again support this point (Figs.7, 8 and 9 in the main text).

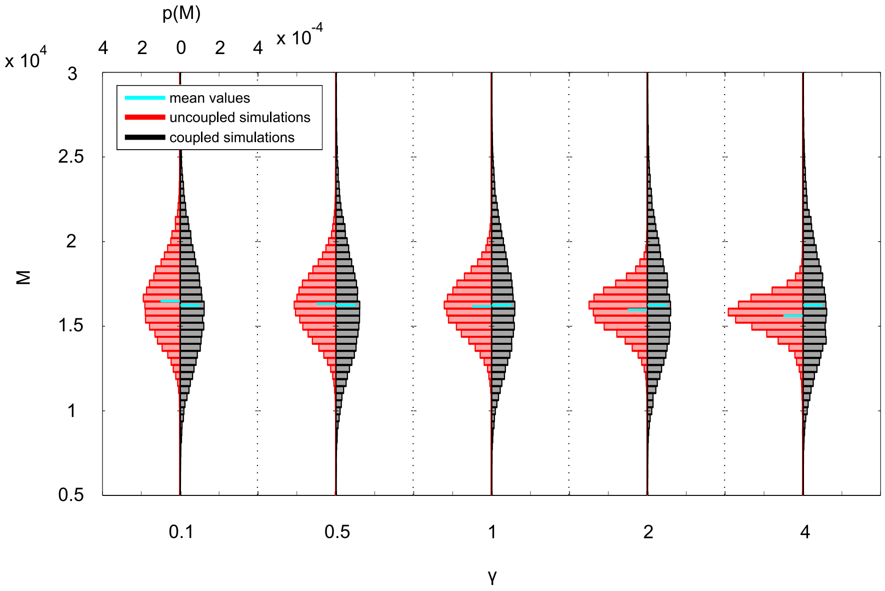

Figure 5. PDFs of both uncoupled and coupled individual niche lineage M, for five different MPCR parameter sets. The death parameters for L and M cells are equal.

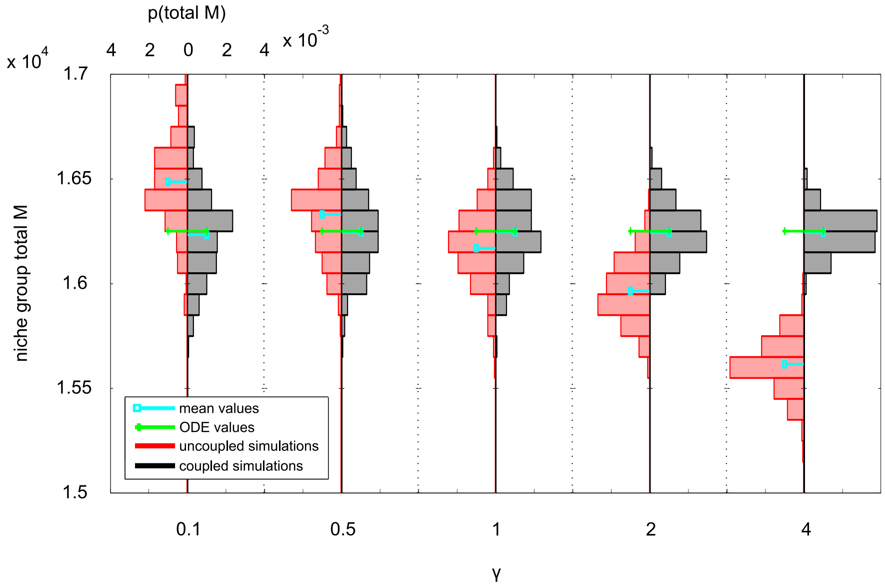

Figure 6. PDFs of both uncoupled and coupled niche group total M, for five different MPCR parameter sets. The ODE values have been marked on, to demonstrate their proximity to the mean of the coupled lineages. The niche group totals are normalised by niche group size. The death parameters for L and M cells are equal.

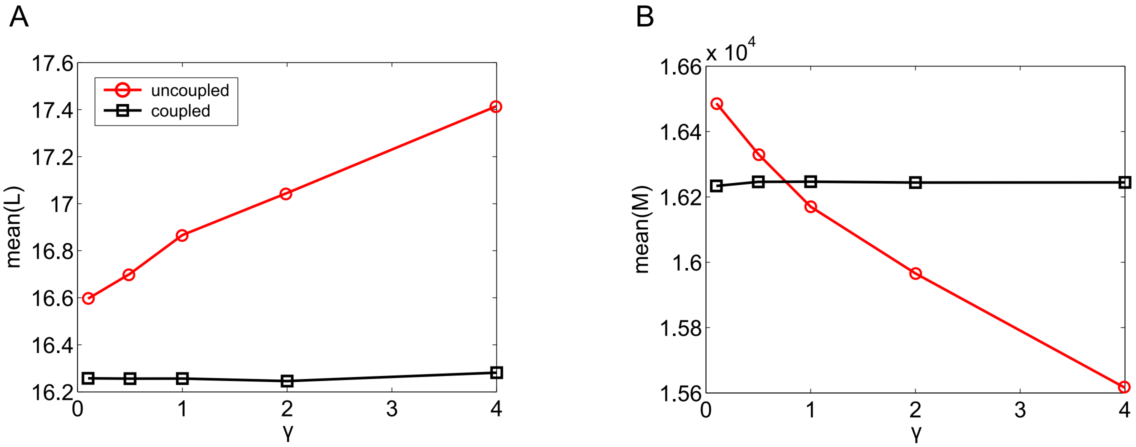

Figure 7. Distribution means of both uncoupled and coupled L and M for various MPCR parameter sets. The death parameters for L and M cells are equal.

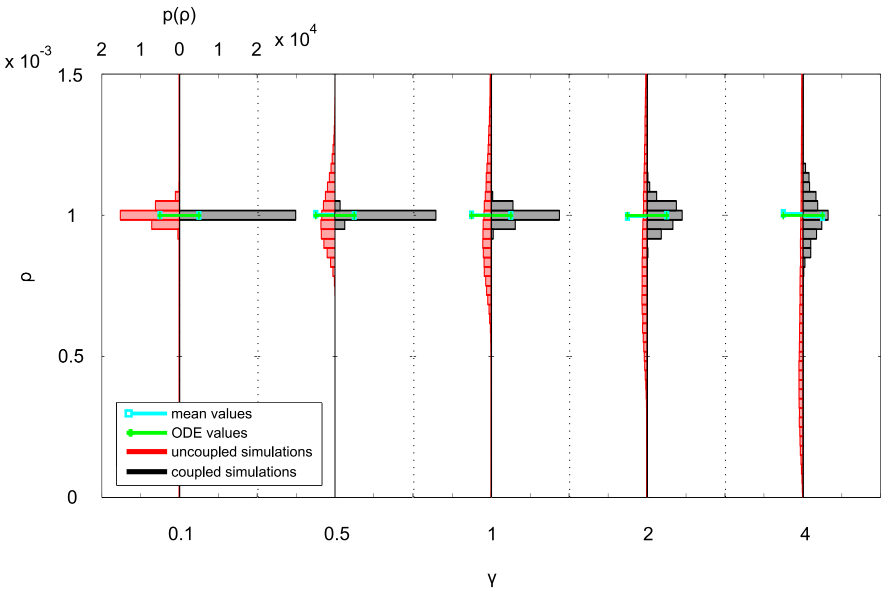

Figure 8. PDFs of both uncoupled and coupled individual niche MPCR, for five different MPCR parameter sets. The ODE values have been marked on, to demonstrate their proximity to the mean of the coupled lineages. The niche group totals are normalised by niche group size. The death parameters for L and M cells are equal.

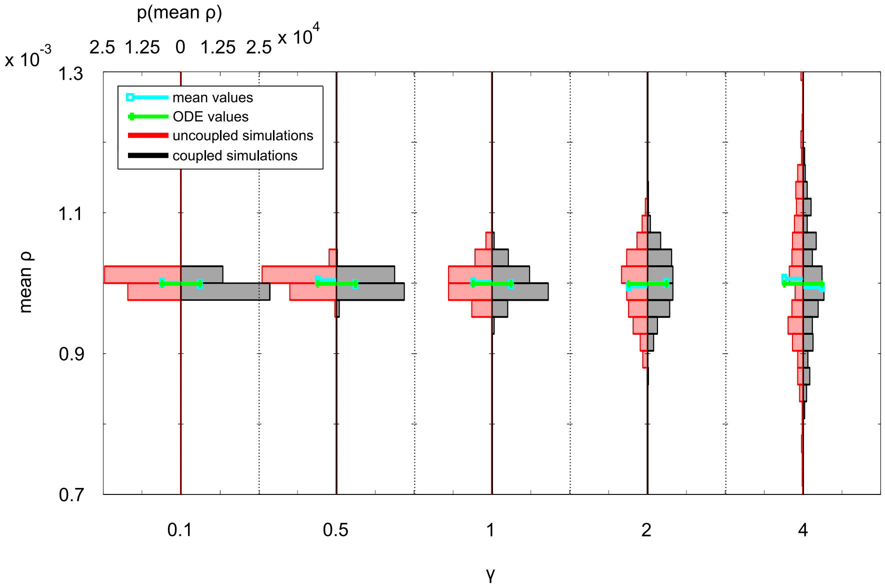

Figure 9. PDFs of both uncoupled and coupled niche group mean MPCR, for five different MPCR parameter sets. The ODE values have been marked on, to demonstrate their proximity to the mean of the coupled lineages. The niche group totals are normalised by niche group size. The death parameters for L and M cells are equal.

References

1. Mangel M, Bonsall MB (2013) Stem cell biology is population biology: differentiation of hematopoietic multipotent progenitors to common lymphoid and myeloid progenitors. Theoretical Biology and Medical Modelling 10: 5.
